# A ROS-responsive, aptamer-targeted graphene oxide nanocomposite for site-specific glutathione release in cerebral ischemia-reperfusion injury

**DOI:** 10.3389/fphar.2025.1543870

**Published:** 2025-05-14

**Authors:** Meiying Li, Lili Wei, Wenxu Liu, Jiawen Wang, Qiujie Lu, Xianjue Chen, Lee Yong Lim, Jingxin Mo

**Affiliations:** ^1^ Lab of Neurology, The Affiliated Hospital of Guilin Medical University, Guilin, China; ^2^ School of Pharmacy, Guilin Medical University, Guilin, China; ^3^ Pharmaceutical Clinical Trial Laboratory, The Affiliated Hospital of Guilin Medical University, Guilin, China; ^4^ School of Clinical Medicine, Guilin Medical University, Guilin, China; ^5^ School of Environmental and Life Sciences, University of Newcastle, Callaghan, NSW, Australia; ^6^ School of Allied Health, University of Western Australia, Perth, WA, Australia; ^7^ Clinical Research Center for Neurological Diseases of Guangxi Province, The Affiliated Hospital of Guilin Medical University, Guilin, China; ^8^ Guangxi Key Laboratory of Big Data Intelligent Cloud Management for Neurological Diseases, Guilin Medical University, Guilin, China; ^9^ Guangxi Engineering Research Center of Digital Medicine and Clinical Translation, Guilin Medical University, Guilin, China

**Keywords:** glutathione, cerebral ischemia-reperfusion injury, graphene oxide, oxidative stress, fibrinogen aptamer

## Abstract

Cerebral ischemia-reperfusion (I/R) injury is a major contributor to mortality and long-term disability worldwide, primarily due to excessive reactive oxygen species (ROS) generation after blood flow is restored. Although current treatments focus on reestablishing perfusion, they offer limited protection against the secondary ROS-mediated injury. Here, we report a multifunctional nanocomposite-graphene oxide loaded with glutathione (GSH) and functionalized with a fibrinogen-targeting aptamer (GO@GSH-FA)-capable of selectively releasing antioxidant cargo within the ischemic brain microenvironment. Characterization revealed a drug-loading capacity of 17.59% ± 3.74% and an entrapment efficiency of 78.78% ± 4.55%, highlighting the robust loading of GSH. The ROS-sensitive borate ester linker ensures that GSH is preferentially liberated in oxidative stress regions, while the fibrinogen aptamer actively targets fibrin-rich thrombotic sites. *In vitro*, GO@GSH-FA significantly restored viability in oxygen-glucose-deprived SH-SY5Y cells (from 31% up to near control levels), reduced inflammatory cytokines, and lowered intracellular ROS. In a Endothelin-1 (ET-1) induced cortical ischemia model, GO@GSH-FA led to a marked decrease in neurological deficit scores (from 7.20 ± 1.16 to 4.20 ± 0.98) and enhanced neuronal survival relative to untreated animals. Collectively, these findings underscore the promise of GO@GSH-FA as a targeted, ROS-responsive platform for mitigating cerebral I/R injury.

## 1 Introduction

Cerebral ischemic stroke remains a critical global health burden, often resulting in severe neurological deficits or death (Zhang M. et al., 2024). While interventions such as thrombolytics and mechanical thrombectomy have revolutionized acute stroke management by restoring blood flow, they also initiate a paradoxical cascade of reperfusion injury. Upon reperfusion, a surge in reactive oxygen species (ROS) occurs, overwhelming endogenous antioxidant defenses and promoting oxidative damage, inflammation, and neuronal death (Freire et al., 2023). This secondary injury phase significantly contributes to poor neurological outcomes, highlighting an urgent need for therapeutic strategies that can attenuate oxidative stress (Silvestro et al., 2024).

**SCHEME 1:**
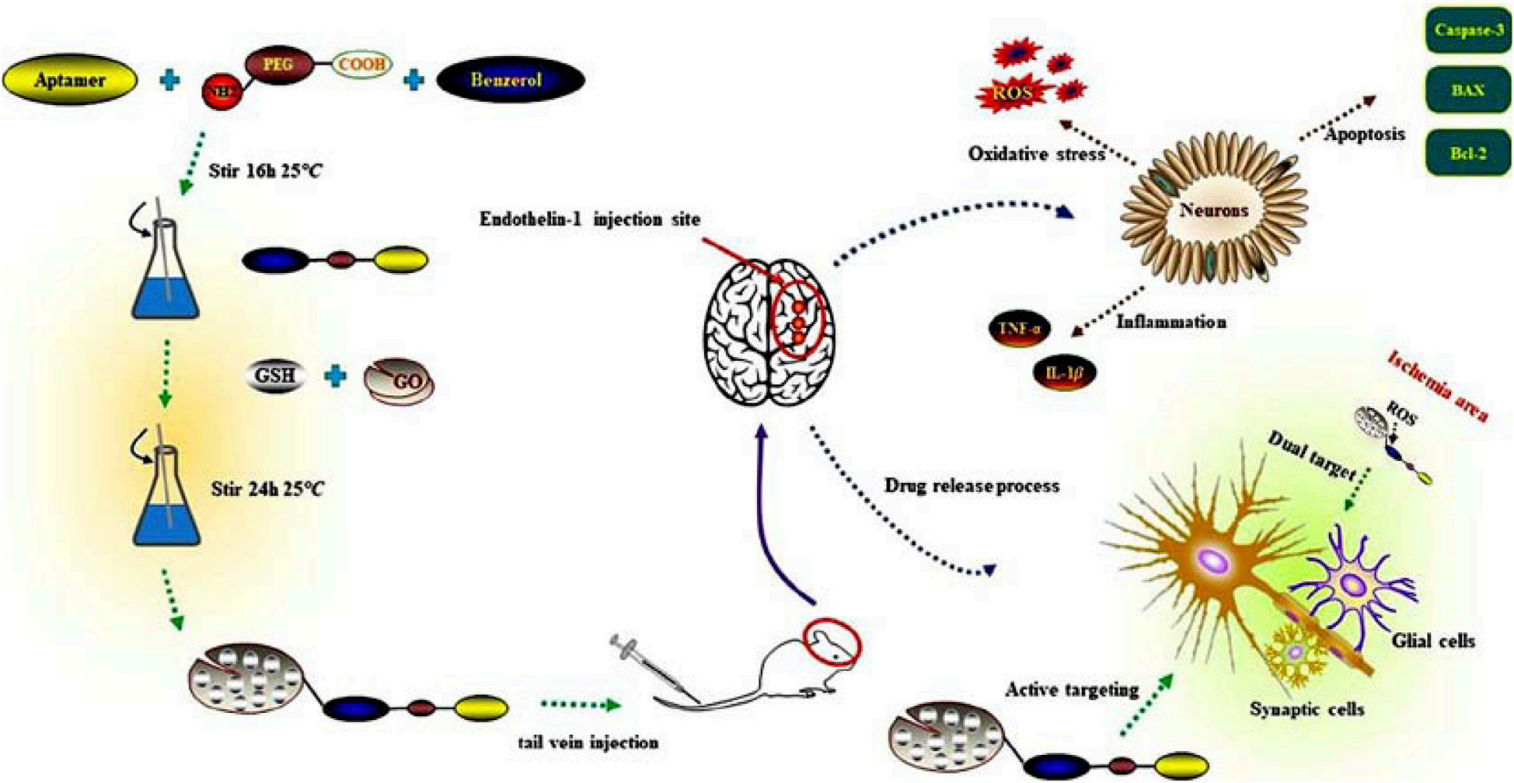
Multifaceted approach to targeting reactive oxygen species in cerebral Ischemia-Reperfusion injury using glutathione-loaded graphene oxide nanoparticles functionalized with fibrinogen aptamer (GO@GSH-FA).

Exogenous antioxidant therapies have shown potential to ameliorate ROS-driven damage. Among these, glutathione (GSH), a tripeptide antioxidant, has demonstrated versatile and potent ROS-scavenging properties (Afzal et al., 2023). However, systemic administration of GSH faces key challenges: rapid metabolic degradation, low bioavailability, and minimal brain targeting (Ezike et al., 2023; Wang et al., 2023; Shen et al., 2022). To overcome these limitations, advanced drug delivery vehicles-particularly nanocarriers-are being explored. Nanotechnology offers the ability to encapsulate and protect fragile therapeutics, enhance permeability across the blood-brain barrier, and enable controlled, stimulus-responsive release at sites of injury (Sun et al., 2024).

Graphene oxide (GO) has emerged as a promising nanoplatform due to its high surface area, rich surface chemistry, and excellent biocompatibility when properly functionalized (Dash et al., 2021). It can be readily conjugated with PEGylated linkers, targeting ligands, and sensitive bonds that respond to specific stimuli (Sun et al., 2023; Battistini et al., 2021). Here, we leverage borate ester bonds to achieve ROS-triggered cleavage and targeted GSH release. Under normal physiological conditions, these borate ester linkages remain stable (Liu et al., 2024). In the oxidative microenvironment of the ischemic brain, however, elevated ROS levels break these bonds, specifically releasing GSH where it is needed most, thereby minimizing off-target effects (Kirchner et al., 2024; Sabatini et al., 2017).

Precise localization of nanocarriers to the injury site is crucial for therapeutic efficacy. Aptamers-short, single-stranded oligonucleotides selected through systematic evolution of ligands by exponential enrichment (SELEX)-offer a powerful, flexible, and stable targeting modality (Xu et al., 2021). Among these, fibrinogen-targeting aptamers have been widely explored for thrombus-specific drug delivery and imaging due to their high affinity for fibrin deposits in ischemic regions (Koudrina et al., 2022). Several studies have demonstrated that fibrinogen aptamers can selectively recognize fibrin clots, enabling targeted delivery of therapeutic agents to thrombotic sites. Koudrina et al. reported that fibrinogen aptamer-functionalized nanoparticles exhibited superior specificity in thrombus imaging, confirming their potential for precision medicine applications in cardiovascular diseases (Koudrina et al., 2022; Koudrina et al., 2020). Similarly, Chen et al. highlighted the efficiency of fibrinogen-binding aptamers in thrombolytic therapies, showcasing their ability to enhance clot dissolution while minimizing systemic side effects (Chen et al., 2022). Unlike antibodies or peptides, aptamers can be synthesized and modified with relative ease, have excellent stability, and avoid issues such as immunogenicity, making them appealing candidates for clinical translation (Brown et al., 2024). In this study, we employ a fibrinogen aptamer (FA) that specifically binds to fibrin deposits commonly found in thrombotic regions post-stroke (Koudrina et al., 2022; Koudrina et al., 2020; Chen et al., 2022). Unlike antibodies or peptides, aptamers can be synthesized and modified with relative ease, have excellent stability, and avoid issues such as immunogenicity, making them appealing candidates for clinical translation (Brown et al., 2024).

We hypothesized that a fibrin-targeted, ROS-responsive GO nanocomposite encapsulating GSH (GO@GSH-FA) would selectively accumulate in ischemic brain regions and release its antioxidant payload in response to local oxidative stress. To test this, we synthesized and characterized the GO@GSH-FA nanocomposite, evaluated its neuroprotective effects in OGD/R cell models, and assessed its therapeutic efficacy in Endothelin-1 (ET-1) induced cortical ischemia models. We examined multiple parameters including oxidative stress markers, pro-inflammatory cytokine levels, apoptotic signaling, and neurological function. Our results underscore the potential of GO@GSH-FA to mitigate the multifactorial challenges posed by I/R injury, representing a significant step forward in targeted nanomedicine for neuroprotection (Scheme 1).

## 2 Materials and methods

### 2.1 Reagents and chemicals

High-purity Glutathione (>98%) and NH_2_-PEG-COOH (A163234) were sourced from Shanghai Aladdin Biochemical Technology Co., Ltd. (Shanghai, China). Graphene oxide (GO) dispersion was acquired from Jiangsu XFNANO Materials Tech. Co., Ltd. (Nanjing, China). Fibrinogen aptamer (5′-NH_2_-GGTTGGTGTGGTTGG-3′) was procured from Ponsure Co., Ltd. (Shanghai, China). All assay kits, except for the CCK-8 assay kit from Dojindo (Kumamoto, Japan), were purchased from Beyotime (Shanghai, China). Analytical-grade monoclonal antibodies against α-tubulin, Bax, Bcl-2, caspase-3, and Iba-1, as well as HRP-conjugated secondary antibodies, were obtained from Abcam (Cambridge, United Kingdom). All other reagents not previously specified are from Aladdin unless otherwise stated.

### 2.2 *In vitro* Cell culture

Human neuroblastoma SH-SY5Y cells were cultured in Dulbecco’s Modified Eagle Medium (DMEM) supplemented with 10% fetal bovine serum and 1% penicillin-streptomycin. Cells were maintained at 37°C in a 5% CO_2_ atmosphere.

### 2.3 Animal models and ethical approval

All animal experiments were conducted in accordance with the guidelines of the Animal Ethics Committee of Guilin Medical University (Ethics Approval No. GLMC-201903037). Male C57BL/6 mice, aged 8–10 weeks and weighing between 20-28 g, were housed under controlled conditions (22°C ± 2°C, 12-hour light/dark cycle) and provided with *ad libitum* access to food and water.

### 2.4 Synthesis and characterization of GO@GSH and GO@GSH-FA nanoparticles

#### 2.4.1 Synthesis of GO@GSH and GO@GSH-FA

For the synthesis of GO@GSH-FA, 110 μg of phenylboronic acid was dissolved in 15 mL of anhydrous DMF under a nitrogen atmosphere to ensure an oxygen-free environment, which is critical for maintaining the integrity of the reactants. Subsequently, 12.12 mg of triethylamine, serving as the base catalyst, were added to the solution, and the mixture was stirred at room temperature for 10 min. This preliminary reaction stage is designed to activate the phenylboronic acid for subsequent conjugation.

Sequentially, 28 μg of amino polyethylene glycol carboxyl (NH_2_-PEG-COOH) and 80 μg of FA-5′NH_2_ (fibrinogen aptamer) were introduced to the mixture. These components are intended to provide the functionality needed for targeting and solubility enhancements in the final nanoparticle complex. After these additions, 4 mg of graphene oxide was added, integrating the backbone of the nanocomplex. The entire mixture was then stirred for 16 h at room temperature to facilitate the coupling reactions.

Following the reaction period, the mixture was centrifuged at 16,904×g for 30 min to separate the unreacted components and byproducts from the target nanoparticles. The supernatant was carefully discarded, ensuring that the precipitate containing the conjugated nanocomplex was retained. This precipitate was subsequently re-dissolved in 15 mL of PBS (pH 7.4) to adjust the physiological relevance of the medium.

**FIGURE 1:**
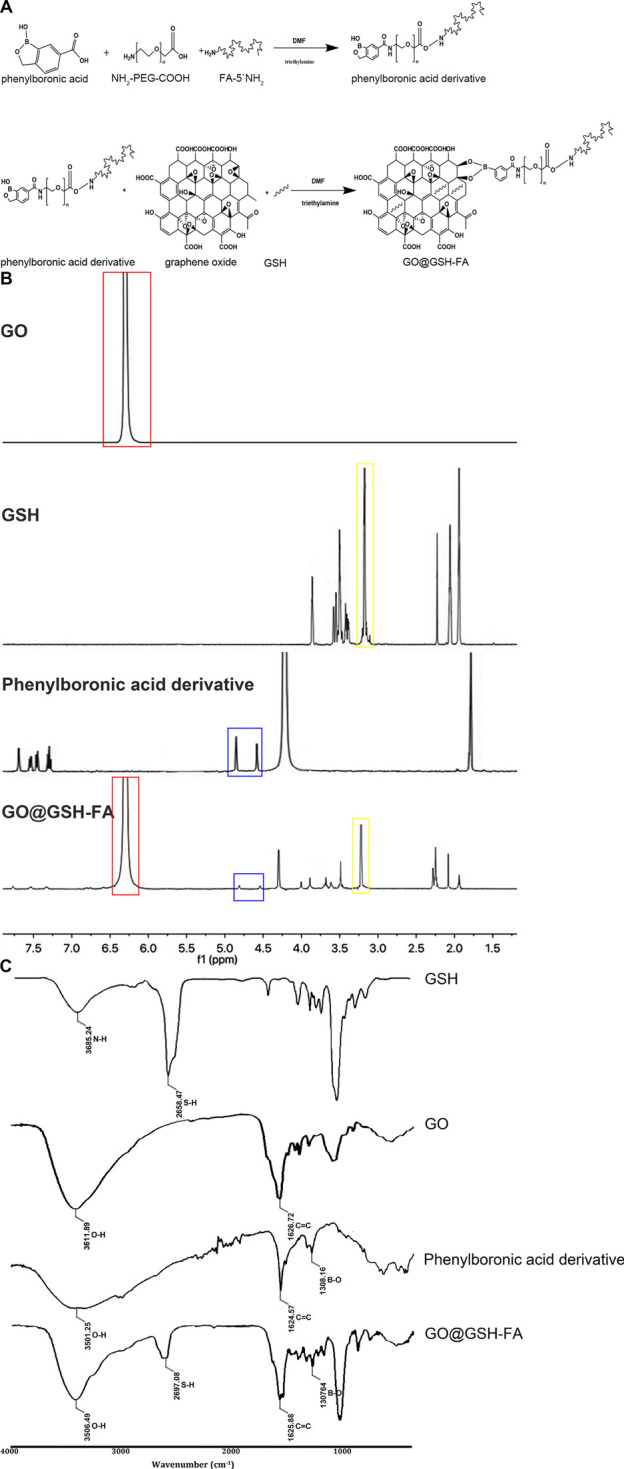
The synthetic pathway of GO@GSH-FA, complemented by its characterization through ^1^H NMR and FTIR spectra. **(A)** Synthetic pathway illustrating the formation of the GO@GSH-FA using click bioorthogonal coupling. This diagram shows the chemical reactions involved in synthesizing the phenylboronic acid derivative and its subsequent conjugation with graphene oxide (GO) and glutathione (GSH) to form GO@GSH-FA. **(B)** ^1^H NMR spectra of GO, GSH, phenylboronic acid derivative, and GO@GSH-FA. The spectra highlight key chemical shifts indicating the presence of aromatic protons in GO (red box), glutathione peaks (yellow box), and phenylboronic acid derivative peaks (blue box). The overlay of these spectra in GO@GSH-FA confirms successful conjugation. **(C)** FTIR spectra of GSH, GO, phenylboronic acid derivative, and GO@GSH-FA. Characteristic absorption bands are marked, showcasing the functional groups present in each component and the successful functionalization of GO@GSH-FA.

To further functionalize the nanoparticles, 20 mg of glutathione (GSH) was added to the solution. Glutathione was chosen for its ability to enhance biocompatibility and cellular uptake. The solution was then stirred for an additional 24 h at room temperature to ensure thorough conjugation. Finally, the resulting solution was freeze-dried, yielding the multi-functional nanocomplex of GO@GSH-FA (Figure 1A).

This synthesis protocol leverages the coupling reaction of FA with graphene oxide through a phenylboronic acid derivative, an approach similar to that described by Li et al. for the functionalization of nanoparticles using click chemistry (Li et al., 2012). The reaction conditions, including the use of DMF as the solvent and triethylamine as the catalyst, are standard in nanoparticle synthesis and are critical for ensuring efficient conjugation of the components (Mohlala et al., 2024).

#### 2.4.2 Characterization of GO@GSH-FA

^1^H NMR spectra were obtained using a NMR-AVANCE NEO 500 MHz spectrometer (Bruker Corporation, Billerica, MA, United States). For analysis, glutathione (GSH), graphene oxide (GO), phenylboronic acid derivative and GO@GSH-FA were dissolved in deuterium oxide (D_2_O).

FTIR spectra were collected using a Thermo 6700 spectrometer (Thermo Fisher Scientific, Waltham, MA, United States). Spectra were recorded in the wavenumber range of 4,000–400 cm^−1^, and the samples were prepared as thin films between two KBr plates.

#### 2.4.3 Physicochemical characterization

Transmission electron microscopy (TEM) was employed to ascertain the particle sizes of GO@GSH and GO@GSH-FA nanoparticles. Zeta potential of GO@GSH and GO@GSH-FA nanoparticles were assessed by dynamic light scattering assay (DLS, Malvern ZSP).

Ultraviolet-visible (UV-Vis) spectroscopy was utilized to quantify drug loading and encapsulation efficiency, based on a pre-established standard curve (R^2^ = 0.9966). The equations for calculating DL (drug loading) and EE (encapsulation efficiency) were as follows:

$$DL\;\left(\%\right)=\frac{amount\;of\;glutathione\;in\;freeze–dried\;nanoparticle\;\left(\mu g\right)}{amount\;of\;freeze–dried\;nanoparticle\;\left(\mu g\right)}\times 100\%$$

$$EE\left(\%\right)=\frac{amount\;ofglutathione\;in\;nanoparticle\;pellet\;\left(\mu g\right)}{amount\;of\;glutathione\;in\;nanoparticle\;dispersion\;\left(\mu g\right)}\times 100\%$$

#### 2.4.4 Nanoparticle stability

The stability of GO@GSH and GO@GSH-FA nanoparticles was rigorously assessed in various media, including phosphate-buffered saline (PBS, pH 7.4) with and without reactive oxygen species (ROS). Samples were incubated at 37°C and periodically analyzed using Dynamic Light Scattering (DLS). DLS was employed to evaluate the zeta potential of the nanoparticles at different time points, thereby assessing their stability and potential for aggregation.

### 2.5 Controlled drug release kinetics

The drug release profiles of GO@GSH and GO@GSH-FA nanoparticles were studied under conditions with and without reactive oxygen species (ROS) to evaluate their ROS-responsive behavior. Nanoparticles were enclosed in dialysis bags (MWCO 12–14 kDa, Spectrum Labs, Rancho Dominguez, CA, United States) and immersed in PBS (Phosphate-Buffered Saline, pH 7.4) containing either 0.5 mM or 5 mM ROS. The system was maintained at 37°C with constant stirring at 140×g using a magnetic stirrer (IKA, Staufen, Germany). At predetermined time intervals, aliquots were withdrawn and replaced with fresh buffer. The concentration of the released drug was quantified using UV-Vis spectroscopy, and the cumulative release percentage was calculated.

### 2.6 *In vitro* cellular model: OGD/R in SH-SY5Y cells

Human neuroblastoma SH-SY5Y cells were cultured in high-glucose Dulbecco’s Modified Eagle Medium (DMEM) supplemented with 10% fetal bovine serum (FBS) (Gibco, Thermo Fisher Scientific, Waltham, MA, United States) and maintained in a 5% CO_2_ incubator at 37°C. An *in vitro* model of cerebral Ischemia-Reperfusion was established using oxygen-glucose deprivation and reoxygenation (OGD/R).

For the OGD phase, cells were washed twice with PBS and then incubated in glucose-free DMEM (Gibco, Thermo Fisher Scientific, Waltham, MA, United States) under hypoxic conditions (1% O_2_, 94% N_2_, 5% CO_2_) in a hypoxia chamber (BioSpherix, Parish, NY, United States) for 8 h. This hypoxic environment was maintained using a gas mixture (1% O_2_, 5% CO_2_, and 94% N_2_).

Following the hypoxia period, the cells were reoxygenated by transferring them back to normoxic conditions (21% O_2_, 5% CO_2_) in a standard cell culture incubator at 37°C. Reoxygenation was carried out in high-glucose DMEM supplemented with 10% FBS for 24 h to mimic reperfusion. This OGD/R model simulates the conditions of cerebral ischemia-reperfusion injury *in vitro*.

### 2.7 Cellular internalization of GO@GSH-FA

SH-SY5Y cells were seeded in six-well plates at a density of 8 × 10^4^ cells per well and cultured for 24 h at 37°C in a 5% CO_2_ atmosphere. Following this, the cells were subjected to oxygen-glucose deprivation and reoxygenation (OGD/R) as described in Section 2.6. Cells were then cultured with various Rhodamine B treatments. Control + Rhodamine B (RB): Cells were treated with 1 µM Rhodamine B in DMEM for 24 h under normoxic conditions. OGD/R + RB: After oxygen-glucose deprivation/reoxygenation (OGD/R), cells were treated with 1 µM Rhodamine B in DMEM for 24 h during the reoxygenation phase. OGD/R + GSH-RB: Cells after OGD/R were treated with 1 µM Rhodamine B alongside glutathione (GSH-RB). The conjugation reaction was performed by incubating 0.1 mg/mL GSH with 1 µM Rhodamine B in PBS for 2 h at room temperature. GO@GSH-RB and GO@GSH-FA-RB: GO@GSH-RB and GO@GSH-FA-RB were prepared by incubating 1 µM Rhodamine B with GSH as described in Section 2.4. After the reoxygenation phase, cells were washed with PBS to remove excess fluorescent compounds. Fluorescence images were captured using a Leica TCS SP5 confocal microscope at excitation/emission wavelengths of 488 nm/525 nm. Quantitative analysis of fluorescence intensity was performed using ImageJ software.

### 2.8 Assessment of Cell viability using CCK-8 assay

Human neuroblastoma SH-SY5Y cells were seeded in 96-well plates at a density of 5 × 10^4^ cells per well and cultured for 24 h. Following this, the cells were subjected to oxygen-glucose deprivation and reoxygenation (OGD/R) as described in Section 2.6. Post-OGD/R treatment, 10 μL of Cell Counting Kit-8 (CCK-8) solution was added to each well and incubated for 1 h at 37°C. The optical density was subsequently measured at a wavelength of 450 nm using a multi-function microplate reader (Molecular Devices, United States). Cell viability was calculated as a ratio of the absorbance in the treated group to that in the control group, which consisted of cells cultured under normal conditions without OGD/R exposure.

### 2.9 Quantification of intracellular ROS levels

SH-SY5Y cells were seeded in six-well plates at a density of 8 × 10^4^ cells per well and cultured for 24 h at 37°C in a 5% CO_2_ atmosphere. Cells were then subjected to various treatments as outlined in Section 2.7. Intracellular reactive oxygen species (ROS) levels were assessed post-treatments using the fluorescent probe 2′,7′-dichlorofluorescin diacetate (DCFH-DA). Cells were incubated with 5 μM DCFH-DA for 30 min at 37°C, followed by three washes with serum-free medium. Fluorescence intensities were measured at excitation/emission wavelengths of 488 nm/525 nm using a Leica TCS SP5 confocal microscope. Data were quantified using ImageJ software and normalized to the control group.

### 2.10 Evaluation of SOD and MDA levels

Following the treatments described in Section 2.9, cells were harvested and subjected to assays for superoxide dismutase (SOD) and malondialdehyde (MDA) using a commercial kit, as per the manufacturer’s guidelines. Protein concentrations were determined using a bicinchoninic acid (BCA) assay kit.

### 2.11 Enzyme-linked immunosorbent assay (ELISA) for cytokine quantification

The levels of pro-inflammatory cytokines TNF-α and IL-1β in the culture supernatant of SH-SY5Y cells were quantified using ELISA kits (R&D Systems, Inc., Minneapolis, United States). Cells were treated as described in Section 2.7. The absorbance was measured at 450 nm using a microplate reader. Cytokine concentrations were calculated based on standard curves and normalized to protein concentrations.

### 2.12 Flow cytometric analysis of apoptosis

Apoptotic cell populations among the various treatment groups were quantified using flow cytometry. SH-SY5Y cells subjected to OGD/R were harvested and resuspended in 100 μL of Binding Buffer. The cells were then stained with 5 μL of fluorescein isothiocyanate (FITC)-labeled Annexin V (20 μg/mL) and incubated in the dark at room temperature for 15 min. Subsequently, 5 μL of propidium iodide (PI; 50 μg/mL) was added to the cell suspension and incubated for an additional 5 min in the dark. Finally, 200 μL of Binding Buffer was added to each sample, which was then immediately analyzed using a FACScan flow cytometer within 1 h of staining.

### 2.13 Western blot analysis for protein expression

SH-SY5Y cells were cultured in six-well plates and subjected to the treatments as described in Section 2.7. Post-treatment, cells were lysed and protein concentrations were quantified using a bicinchoninic acid (BCA) protein assay kit. Equivalent amounts of protein were separated on a 12% SDS-polyacrylamide gel and subsequently transferred onto a polyvinylidene fluoride (PVDF) membrane. The membrane was blocked with 5% non-fat dry milk for 2 h at room temperature. Primary antibodies were applied and incubated overnight at 4°C in Tris-buffered saline with Tween 20 (TBST). Following primary antibody incubation, membranes were washed and incubated with horseradish peroxidase (HRP)-conjugated secondary antibodies for 1 h at room temperature. Protein bands were visualized using enhanced chemiluminescence (ECL) and captured using a ChemiDoc MP imaging system (Bio-RAD, Shanghai, China). Quantitative analysis of protein band intensities was performed using Image Lab software.

For *in vivo* studies, proteins were extracted from the hemispheres of mice subjected to Ischemia-Reperfusion. The extraction and analysis processes followed the same protocol as described for the *in vitro* studies.

### 2.14 Animal model and experimental protocol

#### 2.14.1 Establishment of ischemia-reperfusion mouse model and drug administration protocol

Mice (C57BL/6 strain) were housed under controlled environmental conditions with a 12-hour light/dark cycle and *ad libitum* access to standard rodent chow and water. Prior to surgical procedures, animals were weighed and anesthetized using isoflurane inhalation. Subcutaneous administration of ketoprofen (0.05 mg/10 g body weight) served as an analgesic. A 1 cm^2^ area of dorsal fur was shaved, and the exposed skin was sterilized. A midline dorsal incision (0.8 cm) was made between the Lambda and Bregma sutures. Animals were then positioned in a Cunningham mouse stereotactic frame equipped with a temperature control unit. Cortical injection sites were determined using strain-specific 3D stereotactic maps. Three infusion sites were selected, located 2.0 mm lateral to the midline (ML) and at anteroposterior (AP) coordinates of 0.0, +1.0, and +2.0 mm relative to Bregma. The dorsoventral (DV) depth was set at +1.2 mm from the brain surface. At each site, a 250 μm diameter hole was drilled into the skull, and 1 μL of 1 μg/μL endothelin-1 was injected. The incisions were sutured using 6.0 polyurethane sutures. Post-surgery, animals were placed for 15 min in a recovery chamber maintained at 37°C, followed by a temperature adjustment to 25°C. Two hours post-surgery, mice were administered treatments via caudal vein injection.

The mice were allocated treatments as follows: one group served as healthy controls, another group underwent sham operations, and the remaining mice were randomly divided into three treatment groups following Ischemia-Reperfusion surgery. These treatment groups included a GSH group (0.1 g/kg), a GO@GSH group (equivalent to 0.1 g/kg GSH), and a GO@GSH-FA group (equivalent to 0.1 g/kg GSH).

#### 2.14.2 Criteria for assessing surgical success in the endothelin-1 (ET-1) induced cortical ischemia model

1. Neurological Function Score (Modified Longa System) Neurological deficits were evaluated 1 hour after ET-1 injection using the modified Longa scoring system:
  • 0: No observable neurological deficit (excluded from analysis)
  • 1: Failure to fully extend the left forepaw
  • 2: Consistent circling to the left
  • 3: Falling or leaning to the left side when ambulating
  • 4: No spontaneous movement (severe infarction)

Animals scoring between 1 and 3 were considered to have moderate ischemic deficits and were included in further analyses. Those scoring 0 were excluded for inadequate lesion induction, while those scoring 4 were treated according to humane endpoint guidelines and excluded from the final analysis.

2. Regional Cerebral Blood Flow (rCBF) Monitoring Using Laser Doppler Flowmetry (PeriFlux 5,000, Perimed, Sweden) over the right MCA territory, rCBF was recorded immediately before and after ET-1 injection:
  • Successful Ischemia: A reduction in rCBF to ≤30% of the baseline value within 5 min of ET-1 administration.
  • Partial or Failed Ischemia: An rCBF drop >30% of baseline, indicating insufficient ischemic induction.
3. Reperfusion Assessment
  • Successful Reperfusion: rCBF returning to ≥70% of baseline within 2 h after ET-1 administration.
  • Incomplete Reperfusion: rCBF remaining <70% of baseline beyond the 2-hour mark.
4. Exclusion Criteria
  • Insufficient Ischemia: rCBF reduction remaining above 30% of baseline, indicating failure to achieve the desired level of ischemia.
  • Severe or Absent Deficits: Neurological function scores of 0 or 4 at the one-hour assessment.
  • Excessive Weight Loss: Weight loss exceeding 15% within 24 h post-surgery.
  • Subarachnoid Hemorrhage (SAH): Confirmed via post-mortem examination and deemed incompatible with study objectives.

### 2.15 Behavioral assessments

#### 2.15.1 Open field test (OFT)

Motor and exploratory behaviors were assessed using a square open field apparatus (60 × 60 × 30 cm). Each mouse was individually placed in the arena, and both distance traveled and time spent at rest were recorded over a 10-min period. The apparatus was cleaned between each trial to eliminate olfactory cues.

#### 2.15.2 Neural function defect score (NSS)

Twenty-four hours post-infarction, neurological deficits were scored using the Masao Shimizu Sasamata method (Shimizu-Sasamata et al., 1998). Higher scores indicated more severe neurological impairment.

### 2.16 Immunohistochemical analysis

Mice were humanely sacrificed by deep anesthesia using an overdose of pentobarbital, followed by transcardial perfusion with phosphate-buffered saline (PBS) to clear the blood. Following perfusion, the brains were carefully extracted and fixed in 4% paraformaldehyde overnight. Paraffin-embedded coronal sections (10 μm thickness) were then prepared, with six sections per group. Hematoxylin and eosin staining was employed for general histopathological evaluation, while Nissl staining was used to assess neuronal integrity. For immunofluorescence, sections were blocked with 5% fetal bovine serum (FBS) and incubated overnight with primary antibodies (1:100 dilution) at 4°C. Following washes with phosphate-buffered saline with Tween (PBST), sections were incubated with appropriate secondary antibodies for 2 h at room temperature. Nuclei were counterstained with DAPI for 5 min. Fluorescent images were captured using a Leica DM4B microscope, and at least three images from different animals in each group were quantitatively analyzed. For each experimental group, brain tissue sections were prepared from three animals. In each animal, three coronal sections (10 μm thick) spanning the ipsilateral hippocampal and cortical regions were collected. For each section, five random non-overlapping high-power fields (400× magnification) were selected for quantitative assessment of apoptotic markers (caspase-3, Bax, and Bcl-2) and neuronal integrity (Nissl staining). These fields were chosen to represent different subregions of the ischemic cortex and hippocampus. The average of these five fields was used as a single biological replicate for statistical comparisons.

### 2.17 Statistical analysis

Data are presented as mean ± standard deviation (SD) from at least three independent experiments. Statistical significance was determined by one-way ANOVA with *post hoc* Tukey’s tests or two-way ANOVA as appropriate. Differences were considered significant at p < 0.05.

## 3 Results

### 3.1 Synthesis and characterization of GO@GSH-FA nanoparticles

As illustrated in Figure 1A, phenylboronic acid was conjugated with NH_2_-PEG-COOH and FA-5′NH_2_ to form a phenylboronic acid derivative. This derivative was subsequently linked to glutathione (GSH) loaded graphene oxide (GO) in the presence of triethylamine, resulting in the formation of GO@GSH-FA nanoparticles.

The NMR spectra provided definitive evidence for the chemical environments of the protons and carbons in the synthesized compounds. For GO conjugated with Glutathione (GO@GSH) and GO conjugated with Glutathione and Fibrinogen Aptamer (GO@GSH-FA), characteristic chemical shifts were observed that indicated the successful loading of GSH and conjugation of FA to the GO surface (Figure 1B).

The FTIR spectra further corroborated the NMR findings. Characteristic absorption bands were identified for each compound: GSH displayed bands indicative of its thiol and amine groups; GO showed peaks corresponding to its epoxide and carboxyl groups; and GO@GSH-FA displayed additional bands that were attributed to the presence of FA. These bands confirmed the successful synthesis and functionalization of the nanocomposites (Figure 1C).

### 3.2 Characterization and stability of GO@GSH and GO@GSH-FA nanoparticles

**FIGURE 2:**
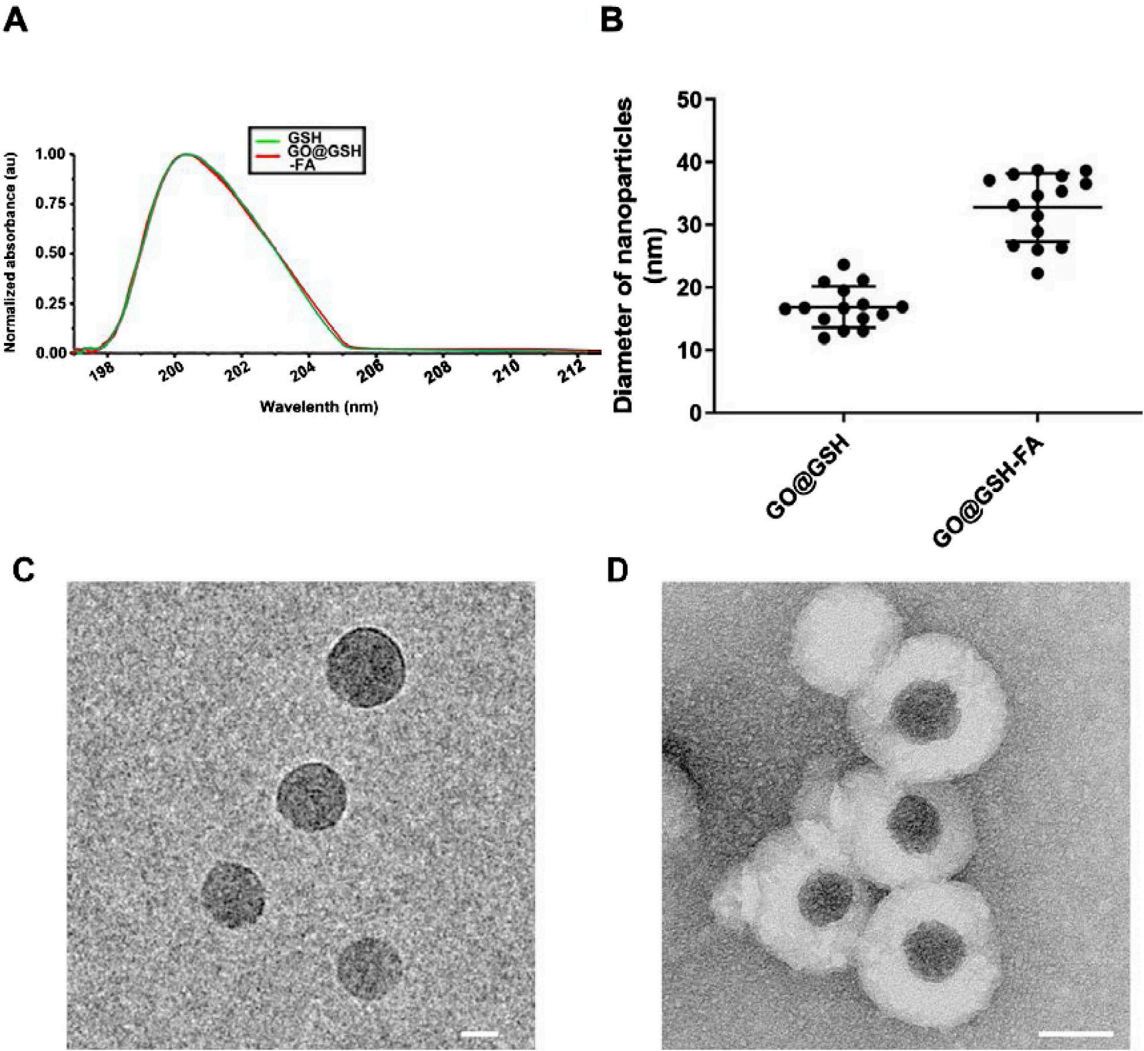
Characterization of GO@GSH and GO@GSH-FA Nanoparticles. **(A)** The UV absorption spectrum of native glutathione (GSH) alongside that of the synthesized GO@GSH-FA nanoparticles, highlighting the characteristic absorption peak of GSH at 200 nm. **(B)** The particle size distribution of GO@GSH and GO@GSH-FA nanoparticles as determined by Dynamic light scattering (DLS). The diameters were measured to be 18 ± 2 nm for GO@GSH and 32 ± 5 nm for GO@GSH-FA. **(C)** TEM Imaging of GO@GSH. The scale bar represents 10 nm. **(D)** TEM Imaging of GO@GSH-FA. The scale bar is set at 20 nm.

The UV-visible absorption spectrum of GO@GSH-FA was analyzed to confirm the presence of glutathione (GSH). A characteristic absorption peak for GSH was observed at 200 nm (Figure 2A), corroborating the successful incorporation of GSH into the nanoparticle structure. Quantitative analyses revealed that GO@GSH-FA nanoparticles were synthesized with a drug entrapment efficiency of 78.78% ± 4.55% and a drug loading capacity of 17.59% ± 3.74%. These metrics underscore the efficiency of the nanoparticle synthesis process.

Dynamic light scattering (DLS) analysis showed that GO@GSH-FA nanoparticles had an average diameter of 32 ± 5 nm, which was larger compared to GO@GSH nanoparticles, which had an average diameter of 18 ± 2 nm (Figure 2B). This significant increase in size suggests successful functionalization with the fibrinogen aptamer. Transmission electron microscopy (TEM) was employed to investigate the morphology of the synthesized nanoparticles. The TEM images revealed that both GO@GSH (Figure 2C) and GO@GSH-FA (Figure 2D) exhibited a thin, sheet-like morphology of the graphene oxide (GO). Additionally, GO@GSH-FA nanoparticles displayed a visible corona structure around GO core, further confirming successful functionalization with the fibrinogen aptamer.

The zeta potential of the nanoparticles was measured to assess their colloidal stability. GO@GSH-FA exhibited a zeta potential of −32 mV, indicating robust colloidal stability and a reduced likelihood of aggregation or precipitation in aqueous conditions. (Serrano-Lotina et al., 2023).

### 3.3 Responsive drug release from GO@GSH-FA nanoparticles

**FIGURE 3:**
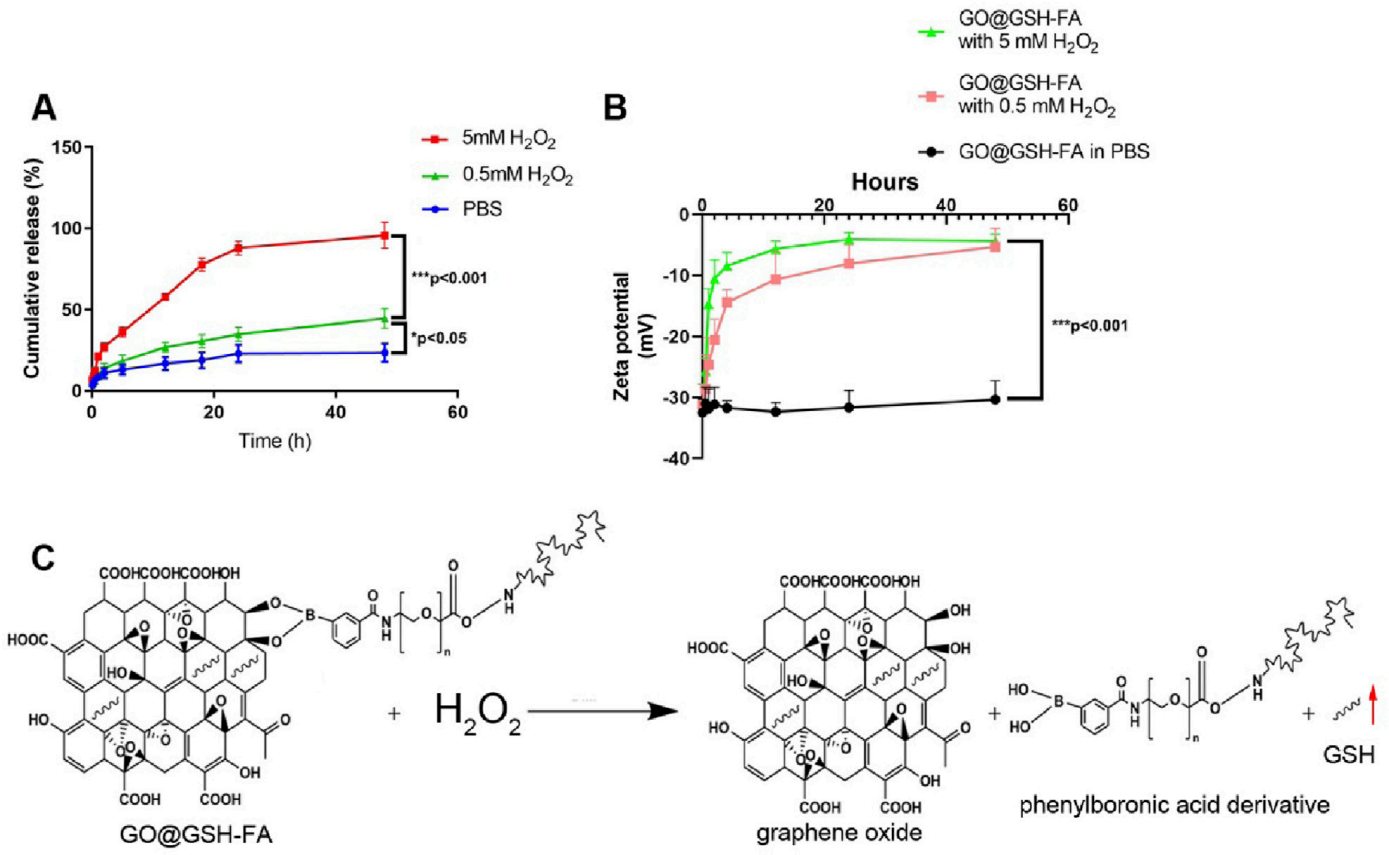
Stability and release profile of GO@GSH-FA. **(A)** Cumulative release of GSH from GO@GSH-FA over 48 h in different conditions. **(B)** Zeta potential measurements of GO@GSH-FA over 48 h in PBS and different concentrations of H_2_O_2_. **(C)** Schematic representation of the oxidative degradation of GO@GSH-FA in the presence of H_2_O_2_, illustrating the release of GSH by detaching the phenylboronic acid derivative. Data are presented as mean ± SD from n = 3 independent samples.

We systematically analyzed the release kinetics of GSH from GO@GSH-FA nanoparticles in the presence of 0, 0.5 and 5 mM H_2_O_2_ to simulate different oxidative stress conditions. As shown in Figure 3A, the cumulative release of GSH was significantly enhanced in the presence of H_2_O_2_, with the 5 mM H_2_O_2_ condition showing the highest release, reaching approximately 100% within 48 h. The release of GSH in the presence of 0.5 mM H_2_O_2_ was higher than in PBS alone (0 mM H_2_O_2_), but lower than in 5 mM H_2_O_2_. This indicates that while 0.5 mM H_2_O_2_ does induce an increased release of GSH compared to the baseline condition, the release rate is still considerably less than that observed at 5 mM H_2_O_2_, demonstrating a dose-dependent response to oxidative stress conditions.

Examining the zeta potential changes, Figure 3B illustrates that GO@GSH-FA nanoparticles exhibited a marked increase in zeta potential over time in both 0.5 mM and 5 mM H_2_O_2_ environments, indicating the oxidative degradation of the nanoparticles. The zeta potential remained stable in PBS, highlighting the oxidative responsiveness of the nanoparticles.

The oxidative degradation mechanism is depicted in Figure 3C, where H_2_O_2_ triggers the breakdown of GO@GSH-FA by detaching the phenylboronic acid derivative, leading to the accelerated release of the GSH. This responsive behavior underscores the potential of GO@GSH-FA nanoparticles for targeted drug delivery in environments with elevated reactive oxygen species (ROS).

### 3.4 Neuroprotective efficacy of GO@GSH-FA on SH-SY5Y cells subjected to OGD/R

**FIGURE 4:**
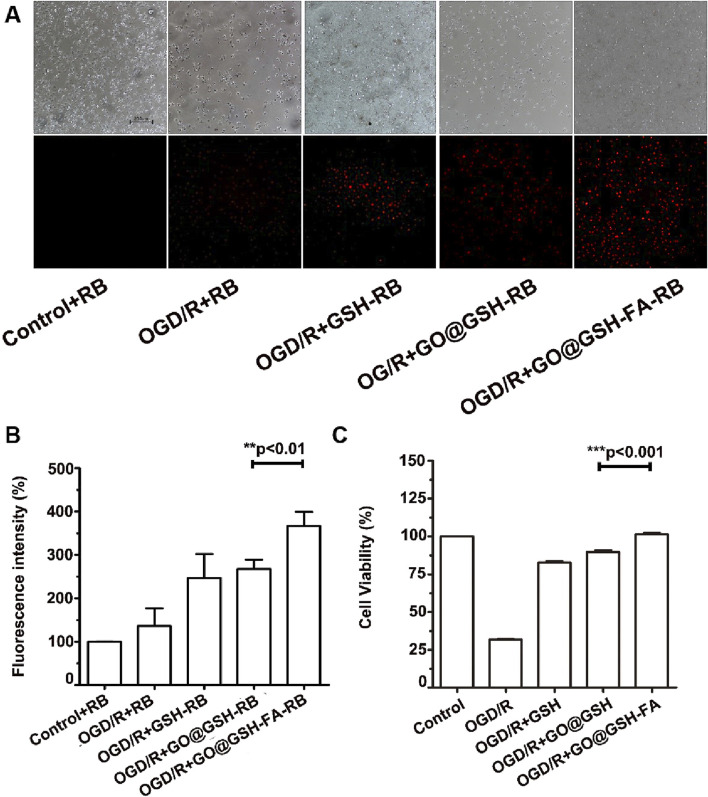
Neuroprotective Effects and Cellular Uptake of GO@GSH-FA in SH-SY5Y Cells Subjected to OGD/R. **(A)** Fluorescence microscopy images depicting the cellular uptake of GO@GSH-FA in SH-SY5Y cells post-OGD/R treatment. Rhodamine B was employed as a fluorescent marker to indicate GO@GSH-FA localization within the cells. Scale bar represents 200 μm. (n = 3). **(B)** Quantitative analysis of fluorescence intensity in SH-SY5Y cells subjected to 8 h of OGD/R and treated with GO@GSH-FA. Data are presented as mean ± SD, (n = 3). Control + Rhodamine B (RB): Cells were treated with 1 µM Rhodamine B in DMEM for 24 h under normoxic conditions. OGD/R + RB: After oxygen-glucose deprivation/reoxygenation (OGD/R), cells were treated with 1 µM Rhodamine B in DMEM for 24 h during the reoxygenation phase. OGD/R + GSH-RB: Cells were treated with 1 µM Rhodamine B conjugated with glutathione (GSH-RB). The conjugation was performed by incubating 0.1 mg/mL GSH with 1 µM Rhodamine B in PBS for 2 h at room temperature. GO@GSH-RB and GO@GSH-FA-RB: GO@GSH-RB and GO@GSH-FA-RB were prepared by incubating 1 µM Rhodamine B with GSH as described in Section 2.1. **(C)** Quantitative analysis of cell viability in SH-SY5Y cells subjected to 8 h of OGD/R and treated with GO@GSH-FA. Data are presented as mean ± SD, (n = 9).

Fluorescence microscopy was employed to assess cellular uptake of GO@GSH-FA. Enhanced uptake was discernible in the Ischemia-Reperfusion + GO@GSH-FA group, as evidenced by increased fluorescence intensity (Figure 4A).

Quantitative analysis of fluorescence intensity revealed a significant increase in uptake across all OGD/R-treated groups compared to the control. The OGD/R + GSH-RB group exhibited a higher fluorescence intensity than the OGD/R + RB group. This enhancement was even more pronounced in the OGD/R + GO@GSH-RB group. The OGD/R + GO@GSH-FA-RB group demonstrated the highest fluorescence intensity, indicating the greatest uptake among all treatment groups (Figure 4B).

Post oxygen-glucose deprivation/reoxygenation (OGD/R), a marked reduction in SH-SY5Y cell viability was observed (Figure 4C). Specifically, cell viability plummeted to 31% ± 0.78% following an 8-hour hypoxic exposure.

To further elucidate the impact of OGD/R on SH-SY5Y cellular morphology, differentiated cells were scrutinized under an optical microscope. Noteworthy morphological aberrations, including pyknosis and a loss of angular morphology, were evident in cells subjected to 8-hour OGD/R treatment.

Remarkably, the GO@GSH-FA treatment group exhibited a significant amelioration in cell viability compared to the OGD/R group, with cell viability restored to levels comparable to the control group, suggesting that the treatment effectively reversed the detrimental effects of OGD/R (Figure 4C).

Collectively, these findings substantiate the neuroprotective potential of GO@GSH-FA, demonstrating its effective cellular uptake and therapeutic efficacy in an *in vitro* model of I/R.

### 3.5 Attenuation of intracellular ROS accumulation and modulation of MDA and SOD levels by GO@GSH-FA

**FIGURE 5:**
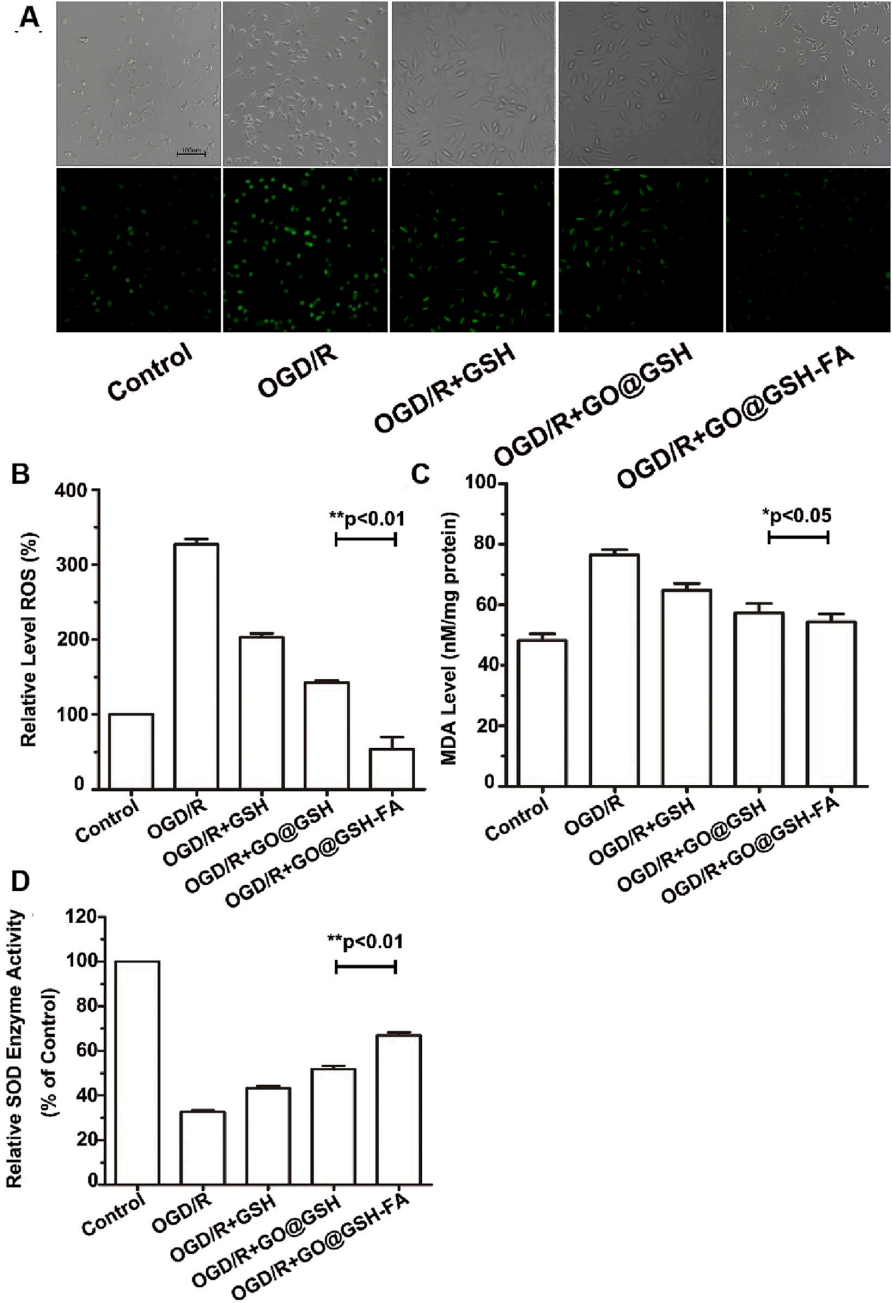
GO@GSH-FA Mitigates Oxidative Stress by Modulating Intracellular ROS, MDA, and SOD Levels. **(A)** Fluorescence micrographs illustrating intracellular ROS levels, visualized using DCFH-DA as a fluorescent probe. Scale bar represents 100 μm. **(B)** Quantitative analysis of reactive oxygen species (ROS) levels in different treatment groups. **(C)** Malondialdehyde (MDA) levels assessed using a commercial assay kit. **(D)** Superoxide dismutase (SOD) activity measured in the various treatment groups. Data are presented as mean ± SD, (n = 3).

Fluorescence micrographs show the intracellular ROS levels in different treatment groups visualized using DCFH-DA as a fluorescent probe (Figure 5A). The control group exhibited low fluorescence, indicating minimal ROS. In contrast, the OGD/R group demonstrated significantly higher fluorescence, indicative of elevated ROS levels. Treatment with GSH and GO@GSH reduced ROS levels, as shown by the decreased fluorescence intensity. The GO@GSH-FA treatment group displayed the lowest fluorescence intensity, suggesting the most substantial reduction in ROS levels among all treatment groups.

As shown in Figure 5B, the quantitative analysis of reactive oxygen species (ROS) levels showed that the OGD/R group had a significant increase in ROS levels compared to the control group. Treatment with GSH and GO@GSH significantly reduced ROS levels compared to the OGD/R group. Notably, the GO@GSH-FA treatment resulted in a further significant reduction in ROS levels, almost restoring them to control levels (p < 0.01).

The assessment of malondialdehyde (MDA) levels revealed that the OGD/R group had significantly higher MDA levels compared to the control group. Treatments with GSH and GO@GSH reduced MDA levels relative to the OGD/R group. Among the treatments, GO@GSH-FA exhibited a significant reduction in MDA levels compared to the OGD/R group, indicating a decrease in lipid peroxidation (p < 0.05) (Figure 5C).

The measurement of superoxide dismutase (SOD) activity showed a significant decrease in the OGD/R group compared to the control group. Treatment with GSH and GO@GSH improved SOD activity compared to the OGD/R group. The GO@GSH-FA treatment resulted in a significant increase in SOD activity, approaching the control levels, demonstrating the most effective restoration of antioxidant enzyme activity (p < 0.01) (Figure 5D).

In summary, the data indicate that GO@GSH-FA significantly mitigates oxidative stress induced by OGD/R by reducing ROS and MDA levels while enhancing SOD activity. These findings suggest that GO@GSH-FA could be a potential therapeutic agent for conditions associated with oxidative stress.

### 3.6 Anti-inflammatory effects of GO@GSH-FA in OGD/R-induced SH-SY5Y cells

To elucidate the potential anti-inflammatory role of GO@GSH-FA in ischemia-induced cellular damage, we quantitatively assessed the levels of key proinflammatory cytokines, namely Tumor Necrosis Factor-α (TNF-α) and, Interleukin-1β (IL-1β) in SH-SY5Y cells subjected to oxygen-glucose deprivation and reoxygenation (OGD/R) and treated with GO@GSH-FA. Enzyme-linked immunosorbent assays (ELISA) were employed for this purpose.

**FIGURE 6:**
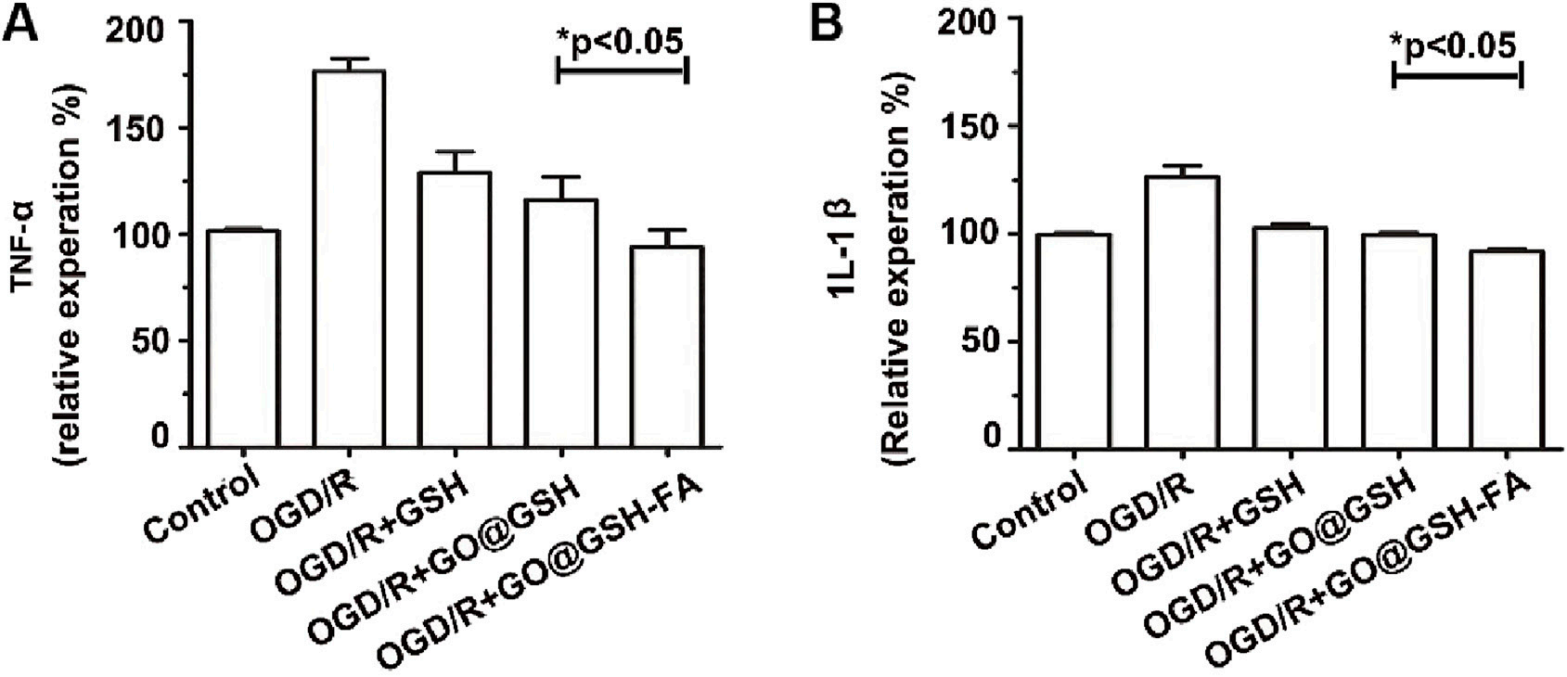
Attenuation of Proinflammatory Cytokine Expression by GO@GSH-FA in OGD/R-Induced SH-SY5Y Cells. The inhibitory effects of GO@GSH-FA on the expression levels of key proinflammatory cytokines, **(A)** Tumor Necrosis Factor-α (TNF-α) and **(B)** Interleukin-1β (IL-1β), in SH-SY5Y cells subjected to oxygen-glucose deprivation and reoxygenation (OGD/R). The cytokine levels were quantitatively assessed using Enzyme-Linked Immunosorbent Assay (ELISA). Data are expressed as mean ± SD, (n = 3).

As depicted in Figure 6, our findings demonstrated a significant downregulation in the expression levels of TNF-α and IL-1β upon treatment with GO@GSH-FA. Specifically, TNF-α levels exhibited a decline of 82.67% (P < 0.05) (Figure 6A), while IL-1β levels were reduced by 34.60% (P < 0.05) (Figure 6B), when compared to the OGD/R-only treated group.

Collectively, these data provide compelling evidence that GO@GSH-FA effectively mitigates the inflammatory response induced by OGD/R, as indicated by the marked reduction in the expression of key proinflammatory markers.

### 3.7 Anti-apoptotic effects of GO@GSH-FA on OGD/R-induced SH-SY5Y cells

**FIGURE 7:**
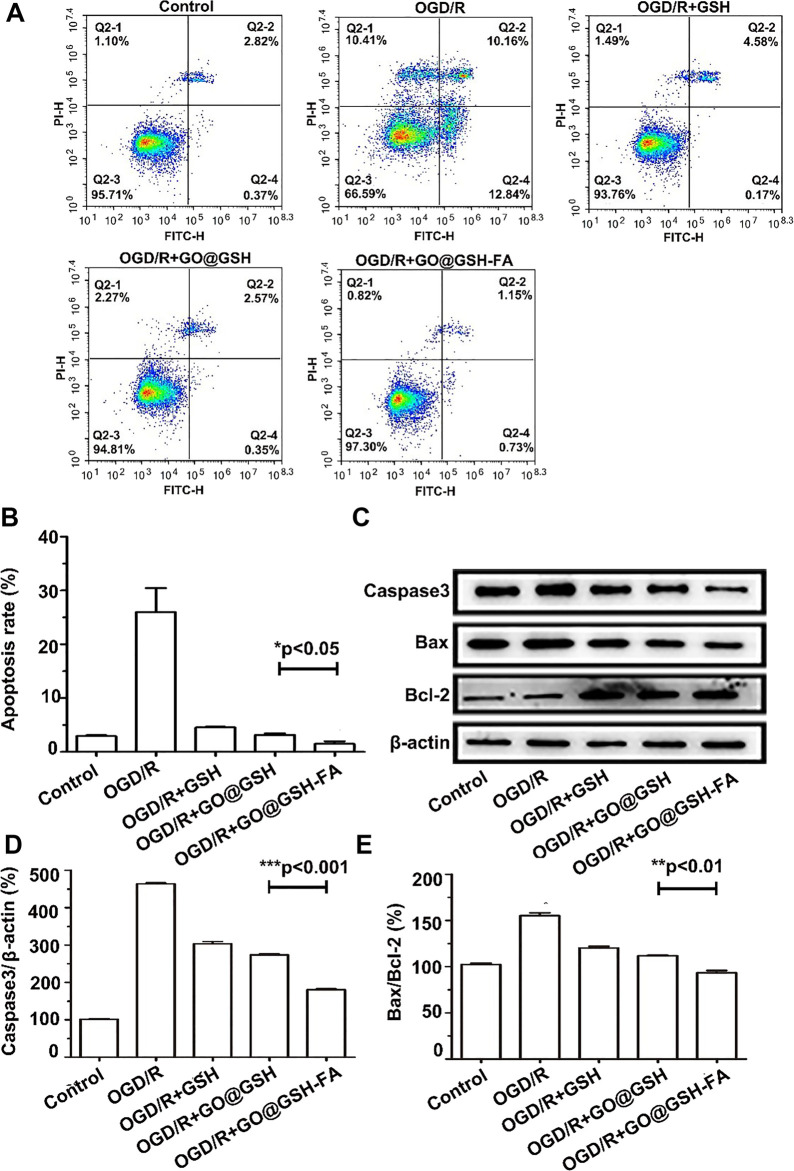
Modulation of Apoptotic Pathways in OGD/R-Induced SH-SY5Y Cells by GO@GSH-FA Treatment. **(A)** Representative flow cytometry images illustrating the extent of apoptosis in SH-SY5Y cells post-OGD/R treatment. Cells were stained with FITC-Annexin V and PI to differentiate between live, early apoptotic, late apoptotic, and necrotic cells. **(B)** Quantitative analysis of apoptosis rates in SH-SY5Y cells subjected to OGD/R and treated with GO@GSH-FA. Data are presented as mean ± SD, (n = 3). **(C)** Western blot analysis depicting the expression levels of key apoptotic proteins, including Bax, caspase-3, and Bcl-2, in the treated cells. β-actin served as a loading control. **(D)** Quantitative analysis of caspase-3 expression relative to β-actin in different treatment groups. Data are presented as mean ± SD, (n = 3). **(E)** Quantitative analysis of Bax/Bcl-2 ratio in different treatment groups. Data are presented as mean ± SD, (n = 3).

To elucidate the mechanistic role of GO@GSH-FA in modulating apoptosis, we investigated the expression profiles of key apoptosis-related proteins. Upon exposure to OGD/R, SH-SY5Y cells exhibited a marked upregulation of the proapoptotic proteins, Bax and caspase-3. Remarkably, treatment with GO@GSH-FA led to a significant downregulation of Bax and caspase-3, while concurrently elevating the expression of the anti-apoptotic protein Bcl-2. These molecular changes were corroborated by enhanced cell viability in the GO@GSH-FA-treated SH-SY5Y cells subjected to OGD/R, as evidenced by flow cytometry assays (Figure 7). Flow cytometric analysis (Figure 7A) indicated that the apoptosis rate was significantly reduced in the GO@GSH-FA-treated group compared to the OGD/R group. Specifically, the OGD/R group showed a high apoptosis rate, whereas the GO@GSH-FA group exhibited a marked decrease in apoptosis, as quantified in Figure 7B. Western blot analysis (Figure 7C) further substantiated these findings. The expression levels of caspase-3 and Bax were significantly lower in the GO@GSH-FA-treated cells, while the expression of Bcl-2 was markedly increased. Quantitative analysis of caspase-3 relative to β-actin (Figure 7D) and the Bax/Bcl-2 ratio (Figure 7E) confirmed these observations, indicating a substantial reduction in apoptotic markers and an increase in anti-apoptotic markers in the GO@GSH-FA treatment group.

Collectively, these results highlight the anti-apoptotic effects of GO@GSH-FA in SH-SY5Y cells subjected to OGD/R, demonstrating its potential as a therapeutic agent for mitigating apoptosis in cerebral Ischemia-Reperfusion injury.

### 3.8 Site-specific accumulation of GO@GSH-FA in cerebral ischemic regions

In the present study, Rhodamine B was employed as a fluorescent marker to track the biodistribution of GO@GSH-FA nanoparticles in C57 mice following intravenous administration. Post-administration, a preferential accumulation of the nanoparticles was observed in the brain tissue. Mice were euthanized 24 h post-injection, and major organs were harvested for analysis.

**FIGURE 8:**
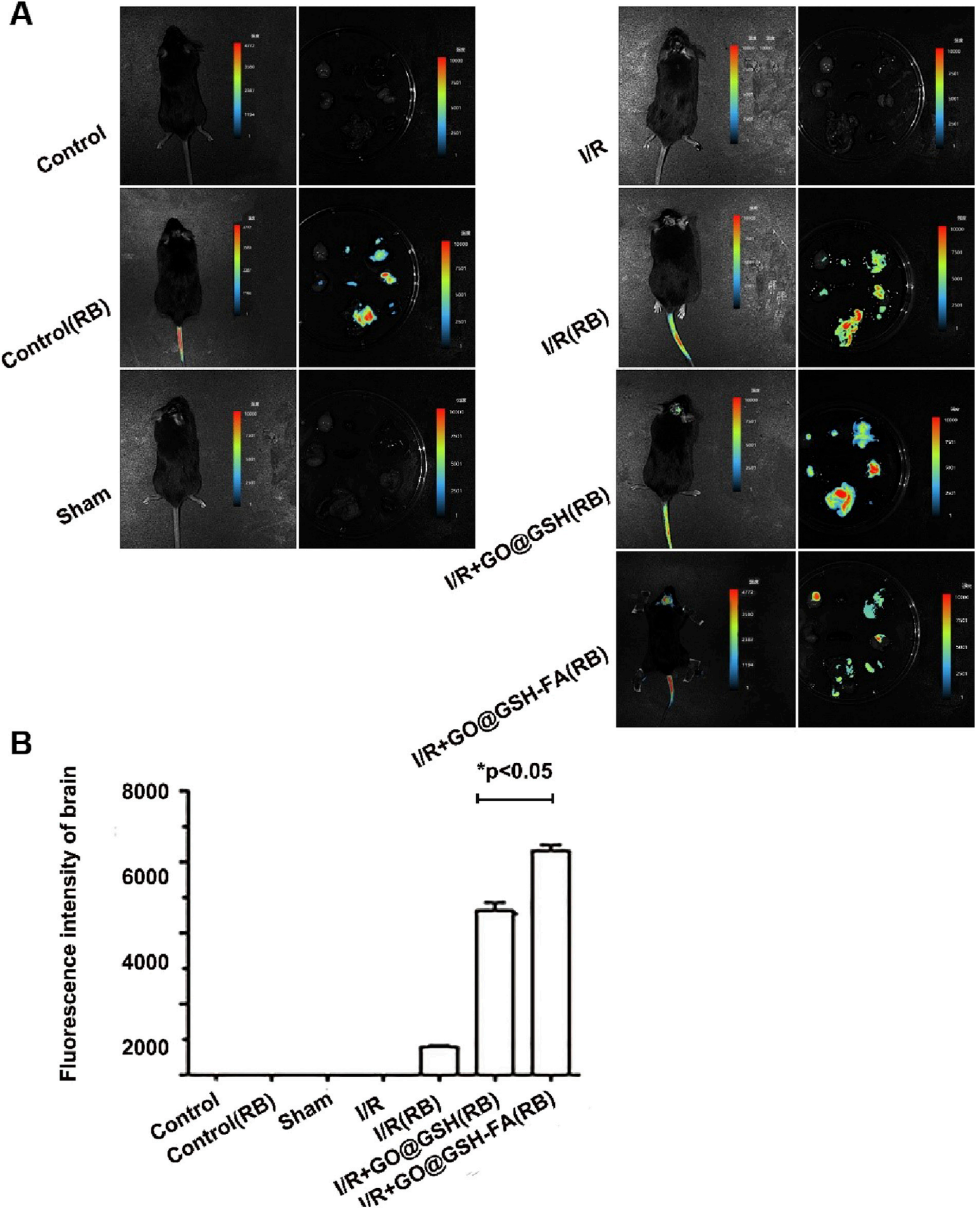
Preferential Accumulation of GO@GSH-FA in Cerebral Ischemic Regions. **(A)** Fluorescence imaging depicting the biodistribution of Rhodamine B-labeled GO@GSH-FA nanoparticles in the brain and other major organs following intravenous administration in C57 mice. **(B)** Quantitative analysis of the fluorescence intensity in brain tissues, providing a comparative assessment across experimental groups. Data are presented as mean ± SD, (n = 6).

Fluorescence imaging demonstrated the biodistribution of Rhodamine B-labeled GO@GSH-FA nanoparticles in the brain and other major organs of C57 mice following intravenous administration (Figure 8A). The no-treatment control group showed no significant fluorescence signal. The treatment control (RB) group, treated with Rhodamine B alone, exhibited minimal fluorescence intensity. In the sham operation group, fluorescence was also negligible, similar to the control groups. In contrast, the Ischemia-Reperfusion group showed increased fluorescence intensity, indicating the accumulation of nanoparticles. The I/R + GO@GSH(RB) group exhibited higher fluorescence in the brain, while the I/R + GO@GSH-FA (RB) group showed the highest fluorescence intensity, suggesting a preferential accumulation of GO@GSH-FA in cerebral ischemic regions.

The quantitative analysis of the fluorescence intensity in brain tissues showed a significant increase in the Ischemia-Reperfusion groups compared to controls. Specifically, the I/R + GO@GSH(RB) group displayed a substantial increase in fluorescence intensity compared to the I/R (RB) group. The I/R + GO@GSH-FA (RB) group exhibited the highest fluorescence intensity among all groups, indicating a significant accumulation of GO@GSH-FA nanoparticles in the brain (p < 0.05) (Figure 8B).

In summary, these results indicate that GO@GSH-FA nanoparticles preferentially accumulate in cerebral ischemic regions, suggesting their potential for targeted delivery in the treatment of ischemic stroke. The significantly higher fluorescence intensity in the I/R + GO@GSH-FA (RB) group demonstrates the enhanced targeting capability of GO@GSH-FA nanoparticles.

### 3.9 Absence of cytotoxic effects in mice treated with GO@GSH-FA

**FIGURE 9:**
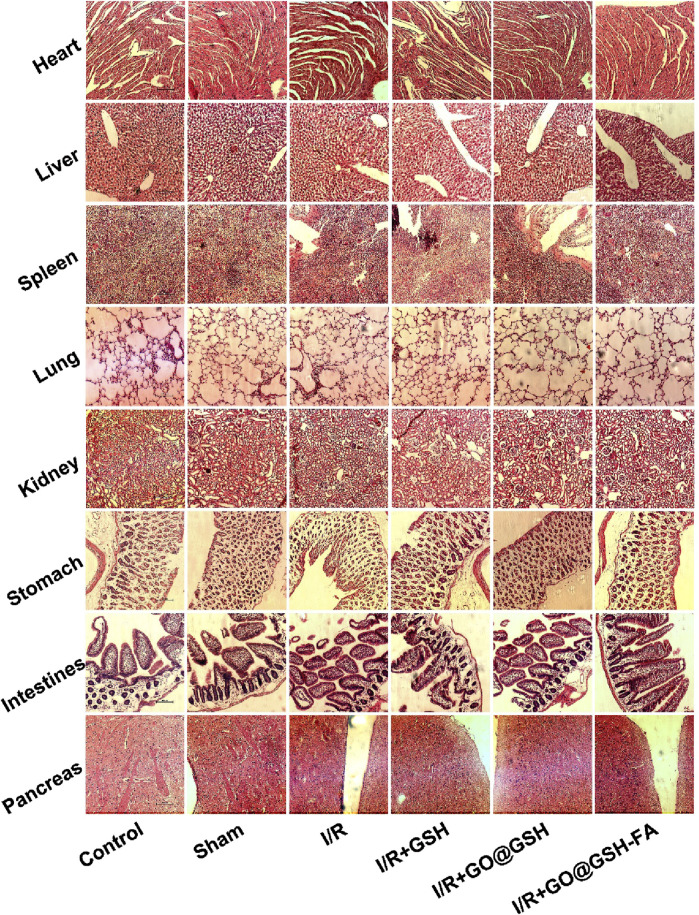
Absence of Cytotoxic Effects in Mice Treated with GO@GSH-FA. Hematoxylin and Eosin (H.E.) stained tissue sections from various organs of mice treated with GO@GSH-FA, serving as an indicator of cytotoxicity. The scale bar represents 100 μm.

Hematoxylin and Eosin (H.E.) stained tissue sections from various organs of mice treated with GO@GSH-FA were examined to assess potential cytotoxic effects (Figure 9). The organs were harvested 2 weeks’ post-treatment, allowing sufficient time for any potential toxic effects to manifest.

The tissue sections of the heart from all groups, including the control, sham, I/R, I/R + GSH, I/R + GO@GSH, and I/R + GO@GSH-FA, showed normal myocardial architecture without any signs of inflammation, necrosis, or other pathological changes.

Liver sections from all groups exhibited normal hepatic architecture with intact hepatocytes and no signs of necrosis, inflammation, or fibrosis. The spleen sections revealed normal splenic architecture across all groups. There were no indications of lymphoid depletion, congestion, or any other abnormalities. Lung tissues from all groups displayed normal alveolar structure without evidence of congestion, edema, or inflammatory cell infiltration. The GO@GSH-FA treated group showed no signs of pulmonary toxicity. Kidney sections from all groups demonstrated normal renal architecture with intact glomeruli and tubules. There were no signs of tubular necrosis, interstitial inflammation, or glomerular damage. The stomach tissues showed normal gastric mucosa, glands, and submucosa in all groups. No pathological changes, such as inflammation or ulceration, were observed.

Intestinal sections from all groups exhibited normal villi and crypt architecture without signs of inflammation, necrosis, or other abnormalities. The pancreas sections revealed normal acinar and islet cell architecture in all groups. There were no signs of inflammation, necrosis, or fibrosis.

In summary, the H&E stained tissue sections from various organs of mice treated with GO@GSH-FA indicated the absence of cytotoxic effects. The normal histological features across all examined organs suggest that GO@GSH-FA does not induce cytotoxicity and is well-tolerated in the treated mice.

### 3.10 Efficacy of GO@GSH-FA in alleviating neurocognitive deficits induced by ischemia-reperfusion

**FIGURE 10:**
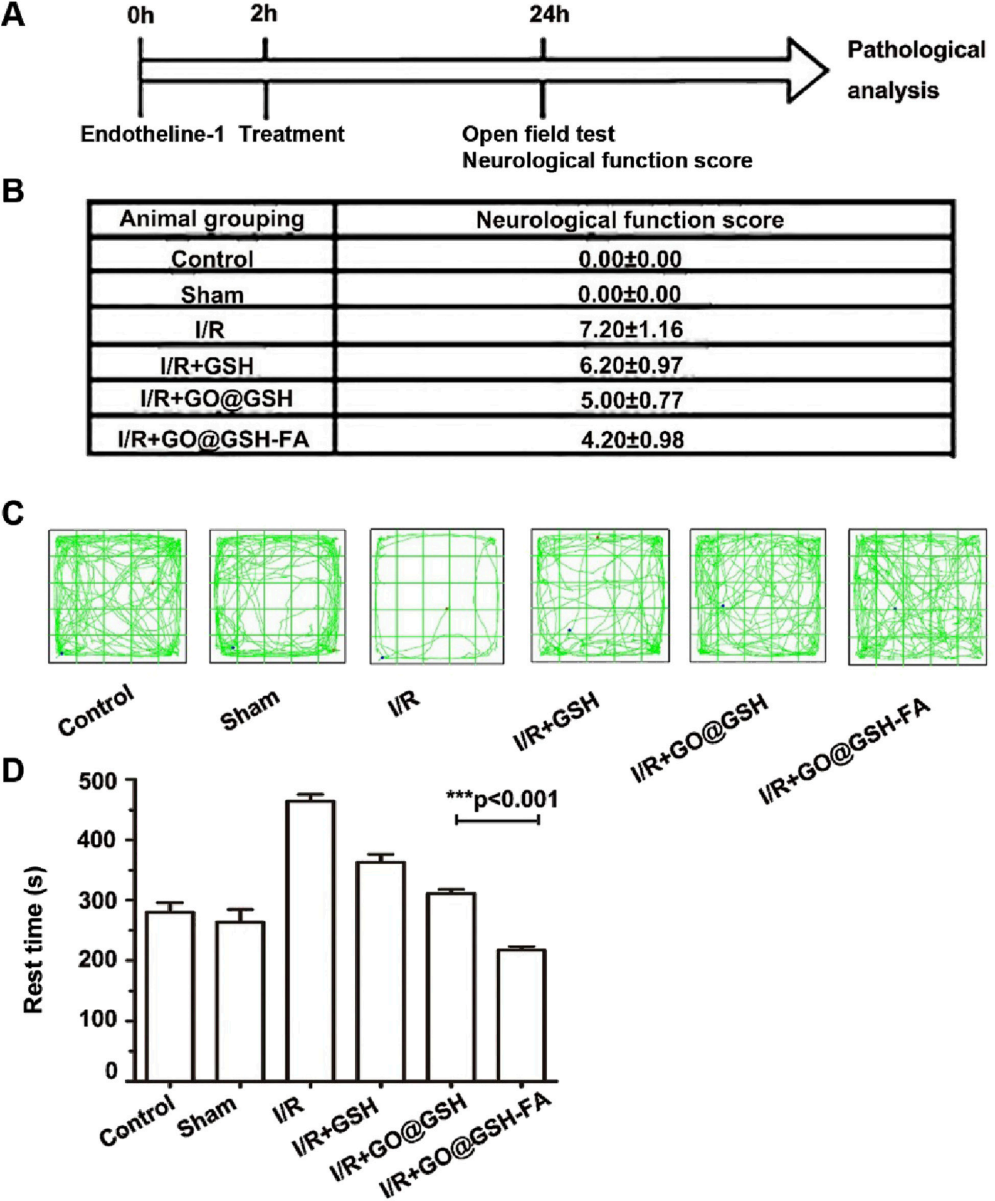
Neuroprotective Effects of GO@GSH-FA on Neurological Deficits and Behavioral Outcomes in Cerebral I/R Model. **(A)** Schematic representation illustrating the experimental design for establishing the Ischemia-Reperfusion model in mice. **(B)** Quantitative analysis of neurological deficit scores in mice subjected to cerebral Ischemia-Reperfusion injury and treated with GO@GSH-FA. Data are presented as mean ± SD, (n = 6). **(C)** Representative tracking paths of mice in the open field test, highlighting alterations in locomotor activity. **(D)** Quantitative assessment of rest time in the open field test for the GO@GSH-FA-treated group. Data are expressed as mean ± SD, (n = 6).

The timeline of the experimental procedure is illustrated in Figure 10A. Endothelin-1 was administered at 0 h to induce ischemia, followed by treatment at 2 h. After 24 h, behavioral assessments including the Open Field Test (OFT) and neurological deficit scoring were performed.

To elucidate the therapeutic potential of GO@GSH-FA in mitigating neurocognitive impairments, comprehensive behavioral assessments were conducted using the Open Field Test (OFT) and Neural Function Defect Score (NSS). Neurological scores were quantitatively evaluated across different experimental groups. In the Ischemia-Reperfusion group, a marked elevation in neurological scores was observed, registering at 7.20 ± 1.16, thereby indicating significant neurological deficits induced by Ischemia-Reperfusion (Figure 10B). In contrast, treatment with GO@GSH-FA led to a substantial reduction in neurological scores, with a decrement to 4.20 ± 0.98.

Representative tracking paths from the OFT (Figure 10C) illustrate these findings. Mice in the Ischemia-Reperfusion group displayed erratic and limited movement patterns, consistent with impaired motor and exploratory behavior. In contrast, mice treated with GO@GSH-FA exhibited more consistent and extensive movement patterns, indicative of preserved neurocognitive function. While the movement patterns in the Ischemia-Reperfusion group appear limited, the erratic nature of their movement, characterized by a lack of purposeful direction, is more indicative of their impaired condition. This erratic behavior, though not fully captured in the static representation of the tracking paths, reflects the reduced and disorganized activity levels observed within the time frame.

Furthermore, the OFT revealed significant differences in the rest times across the experimental groups (Figure 10D). The Ischemia-Reperfusion group exhibited the longest rest time, indicating severe neurocognitive impairments. Conversely, the GO@GSH-FA treatment group showed a significantly reduced rest time, indicating improved neurocognitive function. Specifically, the resting time in the GO@GSH-FA-treated group was statistically shorter compared to both the control group (P < 0.01) and the Ischemia-Reperfusion group (P < 0.001), demonstrating the efficacy of GO@GSH-FA in enhancing neurocognitive outcomes.

Collectively, these results highlight the neuroprotective effects of GO@GSH-FA, demonstrating its potential to mitigate neurological deficits and improve neurocognitive outcomes in an *in vivo* model of cerebral Ischemia-Reperfusion injury.

### 3.11 Neuroprotective efficacy of GO@GSH-FA on neuronal injury post-ischemia-reperfusion

To elucidate the neuroprotective potential of GO@GSH-FA, we performed both histological and molecular analyses on cerebral ischemia-reperfusion injury lesions. Hematoxylin and eosin (H&E) staining, along with Nissl body labeling, were utilized to assess neuronal integrity.

**FIGURE 11:**
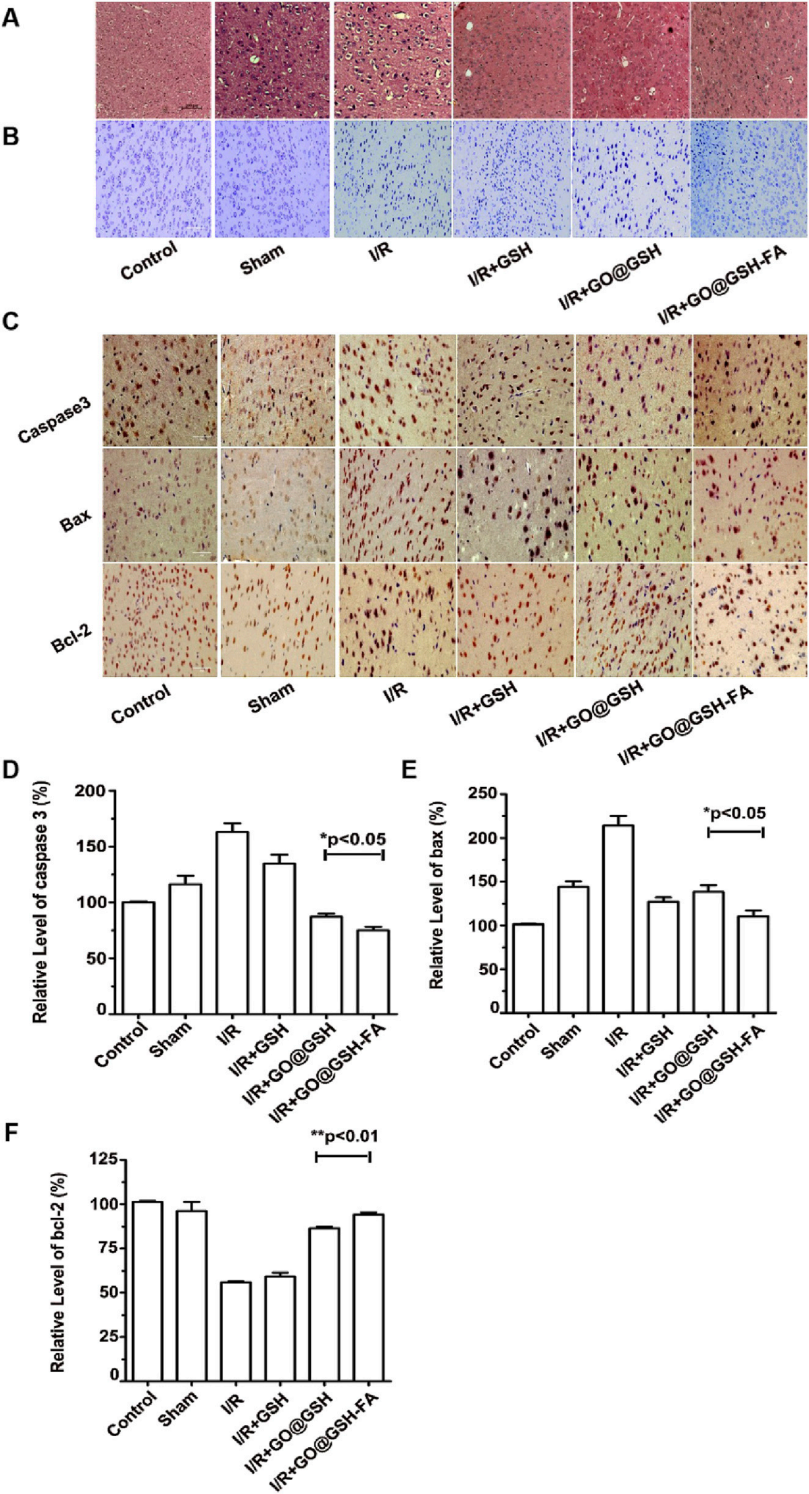
Neuroprotective Effects of GO@GSH-FA on Neuronal Integrity and Apoptosis in Cerebral Ischemia-Reperfusion Injury in Mice. **(A)** Representative hematoxylin and eosin (H&E) stained sections of the hippocampal region, highlighting the morphological changes in neurons. Scale bar: 200 μm; n = 6. **(B)** Nissl staining to visualize Nissl bodies and assess neuronal health. Scale bar: 100 μm; n = 6. **(C)** Immunohistochemical analysis of apoptosis-related proteins, including caspase-3, Bax, and Bcl-2, demonstrating the anti-apoptotic effects of GO@GSH-FA in brain tissue post-cerebral Ischemia-Reperfusion injury. Scale bar: 100 μm; n = 6. **(D)** Quantitative analysis of caspase-3 expression levels in different treatment groups, relative to β-actin. Data are presented as mean ± SD, (n = 3). **(E)** Quantitative analysis of Bax expression levels in different treatment groups, relative to β-actin. Data are presented as mean ± SD, (n = 3). **(F)** Quantitative analysis of Bcl-2 expression levels in different treatment groups, relative to β-actin. Data are presented as mean ± SD, (n = 3).

Histopathological evaluation using H&E staining (Figure 11A) revealed that neurons in the Ischemia-Reperfusion group were disorganized, exhibited contracted and irregular cell bodies, and displayed pyknotic nuclei with indistinct structural features. In contrast, the GO@GSH-FA treatment ameliorated these pathological alterations, as evidenced by a reduction in the number and severity of degenerated, necrotic, and lost neurons. Nissl staining (Figure 11B) showed that the GO@GSH-FA treatment group exhibited a significant increase in Nissl-positive cells compared to the Ischemia-Reperfusion group, indicating better preservation of neuronal integrity. Immunohistochemical assays were conducted to assess the expression of apoptosis-related proteins. The GO@GSH-FA treatment led to a significant downregulation of pro-apoptotic markers Bax and Caspase-3, while upregulating the anti-apoptotic protein Bcl-2. This indicates the anti-apoptotic efficacy of GO@GSH-FA (Figure 11C). Quantitative analysis revealed that the relative level of caspase-3 was significantly lower in the GO@GSH-FA-treated group compared to the Ischemia-Reperfusion group (Figure 11D). The relative level of Bax was also significantly reduced in the GO@GSH-FA-treated group, further confirming the anti-apoptotic effect (Figure 11E). In contrast, the relative level of Bcl-2 was significantly higher in the GO@GSH-FA-treated group, highlighting its neuroprotective and anti-apoptotic properties (Figure 11F).

**FIGURE 12:**
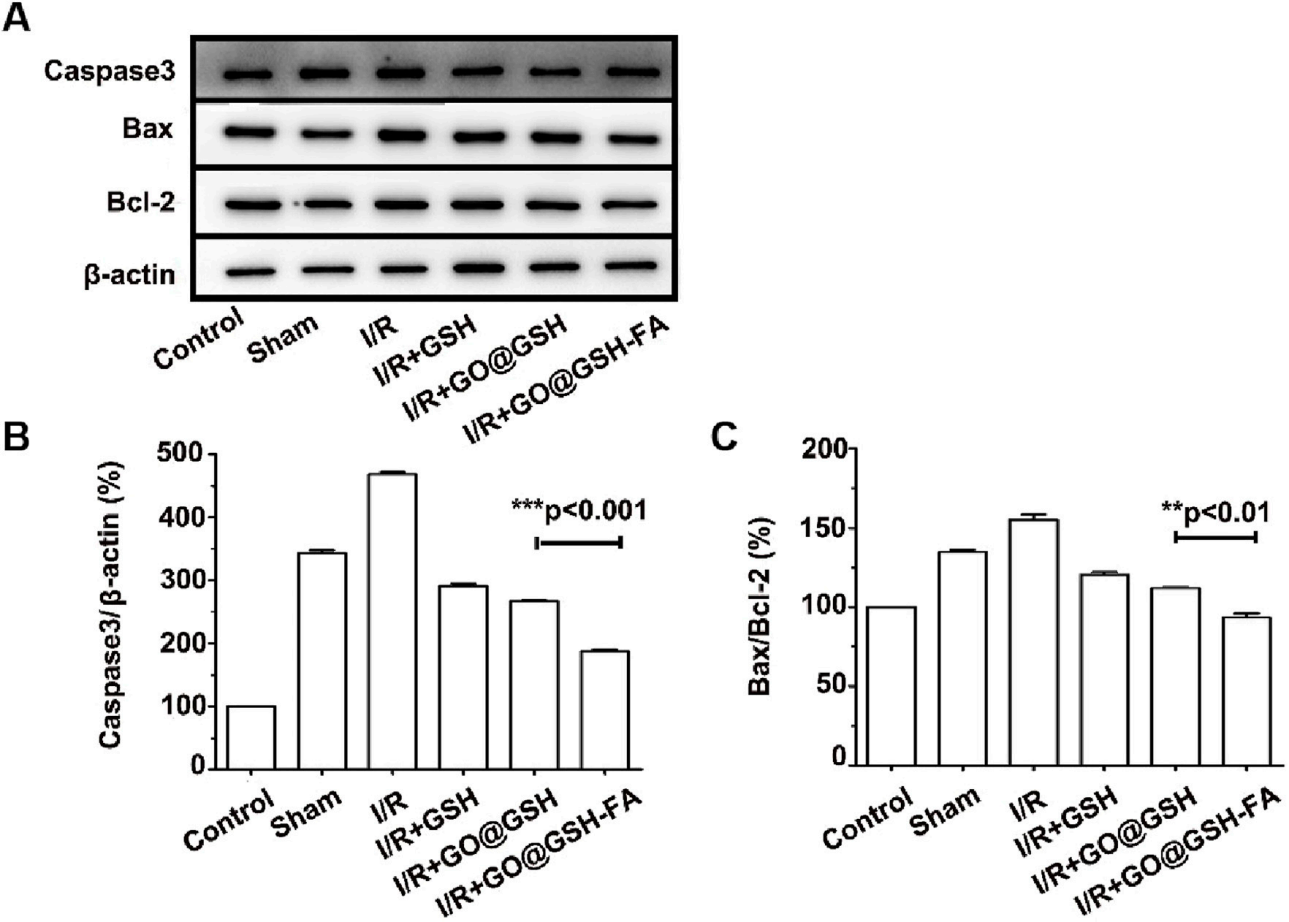
Western blot analyses of apoptosis-related proteins in brain tissue, corroborating the anti-apoptotic effects of GO@GSH-FA post-cerebral Ischemia-Reperfusion injury. **(A)** Western blot analysis depicting the expression levels of key apoptotic proteins, including Bax, caspase-3, and Bcl-2, in the brain tissues. β-actin served as a control. **(B)** Quantitative analysis of caspase-3 expression relative to β-actin in different treatment groups. Data are presented as mean ± SD, (n = 6). **(C)** Quantitative analysis of Bax/Bcl-2 ratio in different treatment groups. Data are presented as mean ± SD, (n = 6).

Western blot analysis depicting the expression levels of key apoptotic proteins including Bax, caspase-3, and Bcl-2 in the brain tissues. β-actin served as a control (Figure 12A). To ensure transparency and address the completeness of the data presentation, representative full-membrane images have been included in the Supplementary File accompanying this paper. Quantitative analysis of caspase-3 expression relative to β-actin in different treatment groups. The GO@GSH-FA-treated group showed a significant reduction in caspase-3 levels compared to the Ischemia-Reperfusion group (Figure 12B). Quantitative analysis of the Bax/Bcl-2 ratio in different treatment groups. The GO@GSH-FA-treated group exhibited a significantly lower Bax/Bcl-2 ratio, indicating a shift towards anti-apoptotic signaling (Figure 12C).

Collectively, these findings indicate that GO@GSH-FA effectively preserves neuronal integrity and reduces apoptosis in a cerebral Ischemia-Reperfusion injury model, demonstrating its potential as a therapeutic agent for neuroprotection.

## 4 Discussion

Our findings underscore the potential of GO@GSH-FA nanoparticles as a cutting-edge, precision therapeutic strategy for mitigating cerebral ischemia-reperfusion (I/R) injury. Through a series of *in vitro* and *in vivo* experiments, we demonstrate that these nanoparticles not only attenuate pathological features-such as oxidative stress, inflammation, and apoptosis-but also appear to do so via targeted and ROS-sensitive mechanisms. This aligns with, yet clearly advances, the current landscape of nanoparticle-based interventions for I/R injury and neurological disorders.

Cerebral I/R injury remains a leading cause of morbidity and mortality, largely due to exacerbated oxidative stress and inflammation following reperfusion (Ye et al., 2025; Zhang C. et al., 2024). Although nanoparticle platforms have previously shown promise in enhancing drug delivery and reducing oxidative damage, the present study is among the first to detail a nanoparticle construct that offers both selective targeting of ischemic tissue and context-dependent release of therapeutic cargo (Zhang C. et al., 2024; Xu et al., 2020; Huang et al., 2022). By leveraging the fibrin-targeting specificity of our formulation-an attribute informed by the well-characterized upregulation of fibrin and associated inflammatory markers in acute ischemic conditions (Falanga et al., 2022) -we have engineered a platform that localizes precisely to damaged cerebral vasculature. The subsequent ROS-responsive bond cleavage of boric acid-modified graphene oxide assures that our nanoparticles function as “smart” carriers, releasing their therapeutic payload predominantly in disease-relevant microenvironments.

Critically, when contrasted with earlier nanoparticle designs, the GO@GSH-FA system incorporates notable advancements. Previous efforts using cerium oxide nanoparticles and other inorganic platforms demonstrated commendable antioxidant capabilities but often lacked robust targeting mechanisms or detailed mechanistic insight (Nag et al., 2024; Jeevanandam et al., 2022; Attarilar et al., 2020). Our study addresses these gaps, reinforcing the notion that effective therapy in the context of cerebral I/R injury is not solely contingent on antioxidative potency but equally on precise localization and on-demand payload release. This synergy likely contributes to the significant reduction in pro-inflammatory markers and a more pronounced attenuation of neuronal cell death in our models.

While these findings are promising, several considerations merit further inquiry. Our animal models, induced using endothelin-1 (ET-1), reflect clinically relevant cerebral I/R conditions yet may not fully capture the complexity of human pathophysiology, including inter-individual anatomical variation and comorbidities (Hougaard et al., 2020). It remains essential to establish the long-term safety profile and assess the fate of GO@GSH-FA nanoparticles *in vivo*, including their potential off-target effects and clearance pathways. As the next phase of this research, more sophisticated *in vivo* models, incorporating chronic injury paradigms, long-term follow-ups, and genetic or pharmacological modifications of inflammatory pathways, will be critical to substantiate the translational potential of this approach.

Moreover, the interplay between nitric oxide (NO) signaling and the observed therapeutic outcomes is of particular interest. NO is intricately involved in both vascular homeostasis and inflammatory processes (Tran et al., 2022). Future investigations will be directed toward dissecting how NO bioavailability and signaling intersect with the mechanisms triggered by GO@GSH-FA nanoparticles, potentially guiding further refinements in nanoparticle design and functionalization.

In conclusion, our results provide a compelling foundation for the use of targeted, ROS-responsive GO@GSH-FA nanoparticles as a therapeutic platform in cerebral I/R injury. The synergy of molecular targeting, responsive release, and antioxidative potential represents a significant conceptual leap forward. Moving ahead, refining these nanoparticles, validating their efficacy across diverse experimental and clinical settings, and unraveling the nuanced molecular interplay with endogenous signaling pathways will be paramount. Through these efforts, we envisage a future in which the precision and adaptability of nanomaterials revolutionize treatment strategies for ischemic stroke and related cerebrovascular pathologies.

## 5 Conclusion

Our findings highlight the potential of a multifunctional, aptamer-targeted, ROS-responsive GO nanocomposite to effectively mitigate cerebral I/R injury. By delivering GSH specifically at sites of elevated oxidative stress, GO@GSH-FA significantly reduced infarct size, improved neurological function, and enhanced neuronal survival *in vivo*. While these results support the potential of this platform for targeted antioxidant therapy, further investigations are required to optimize its formulation, evaluate long-term safety, and assess translational feasibility.

## Data Availability

The original contributions presented in the study are included in the article/Supplementary Material, further inquiries can be directed to the corresponding author.
