# Supplementary material for: A ROS-responsive, aptamer-targeted graphene oxide nanocomposite for site-specific glutathione release in cerebral ischemia-reperfusion injury
Source: Front Pharmacol. 2025 May 14;16:1543870. doi: 10.3389/fphar.2025.1543870 (PMC12116469; doi:10.3389/fphar.2025.1543870)

Supplementary file

A ROS-Responsive, Aptamer-Targeted Graphene Oxide Nanocomposite for Site-Specific Glutathione Release in Cerebral Ischemia-Reperfusion Injury

Meiying Li^1,2†^, Lili Wei^3†^, Wenxu Liu^2^, Jiawen Wang^1^, Qiujie Lu^9^, Xianjue Chen^4^, Lee Yong Lim^5^, Jingxin Mo^1,6,7,8^*

^1^Lab of Neurology, The Affiliated Hospital of Guilin Medical University, Guilin, China, ^2^School of Pharmacy, Guilin Medical University, Guilin, China,

^3^Pharmaceutical Clinical Trial Laboratory, The Affiliated Hospital of Guilin Medical University, Guilin, China,

^4^School of Environmental and Life Sciences, University of Newcastle, Callaghan, New South Wales 2308, Australia

^5^School of Allied Health, University of Western Australia, Perth, Western Australia 6009, Australia,

^6^Clinical Research Center for Neurological Diseases of Guangxi Province, The Affiliated Hospital of Guilin Medical University, Guilin, China,

^7^Guangxi Key Laboratory of Big Data Intelligent Cloud Management for Neurological Diseases, Guilin Medical University, Guilin, China,

^8^Guangxi Engineering Research Center of Digital Medicine and Clinical Translation, Guilin Medical University, Guilin, China,

^9^School of Clinical Medicine, Guilin Medical University, Guilin, China.

*Correspondence: Jingxin Mo, jingxin.mo@hotmail.com

†These authors have contributed equally to this work.

Representative full-membrane images of Figure 12 (A):

Caspase3


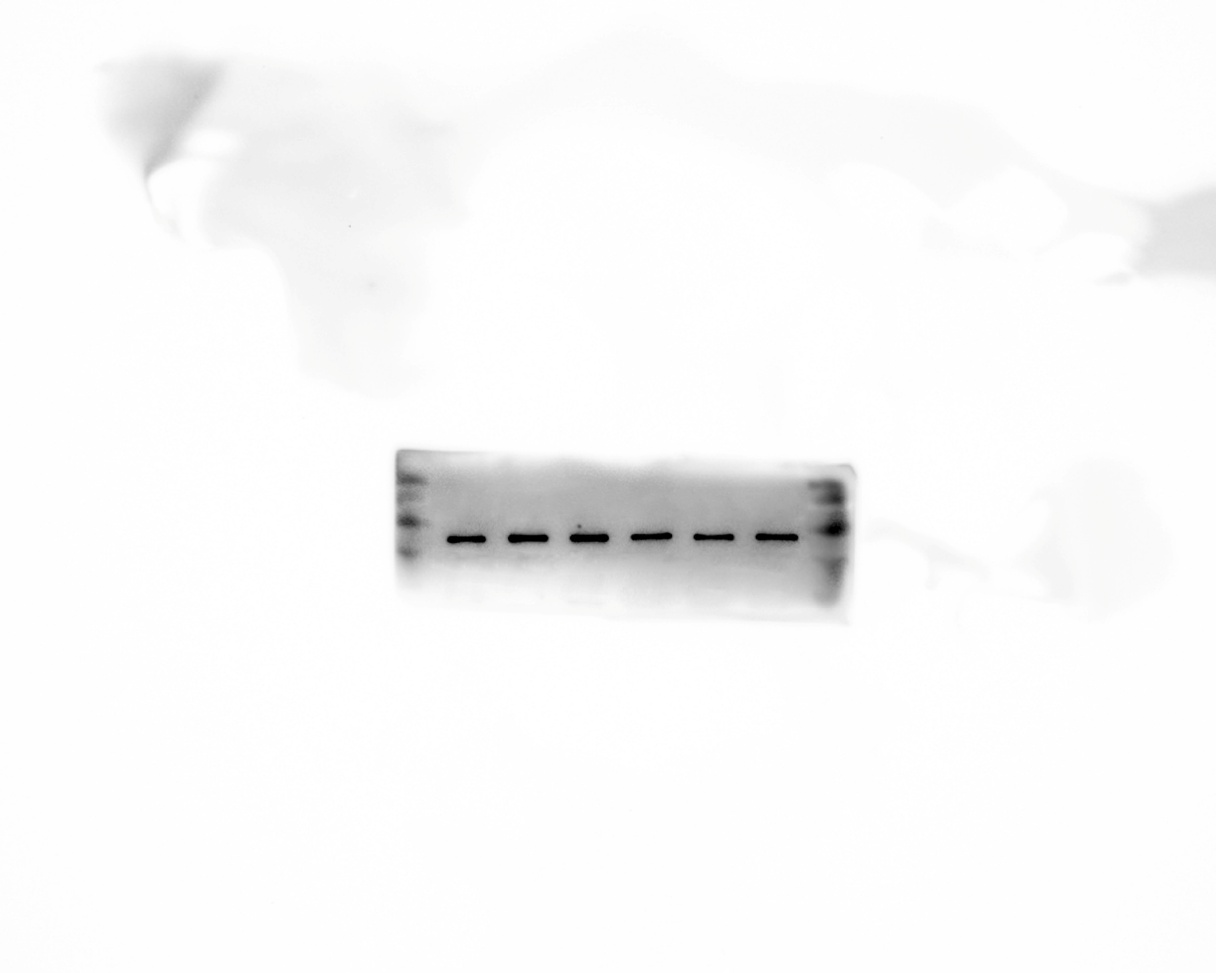


Bax


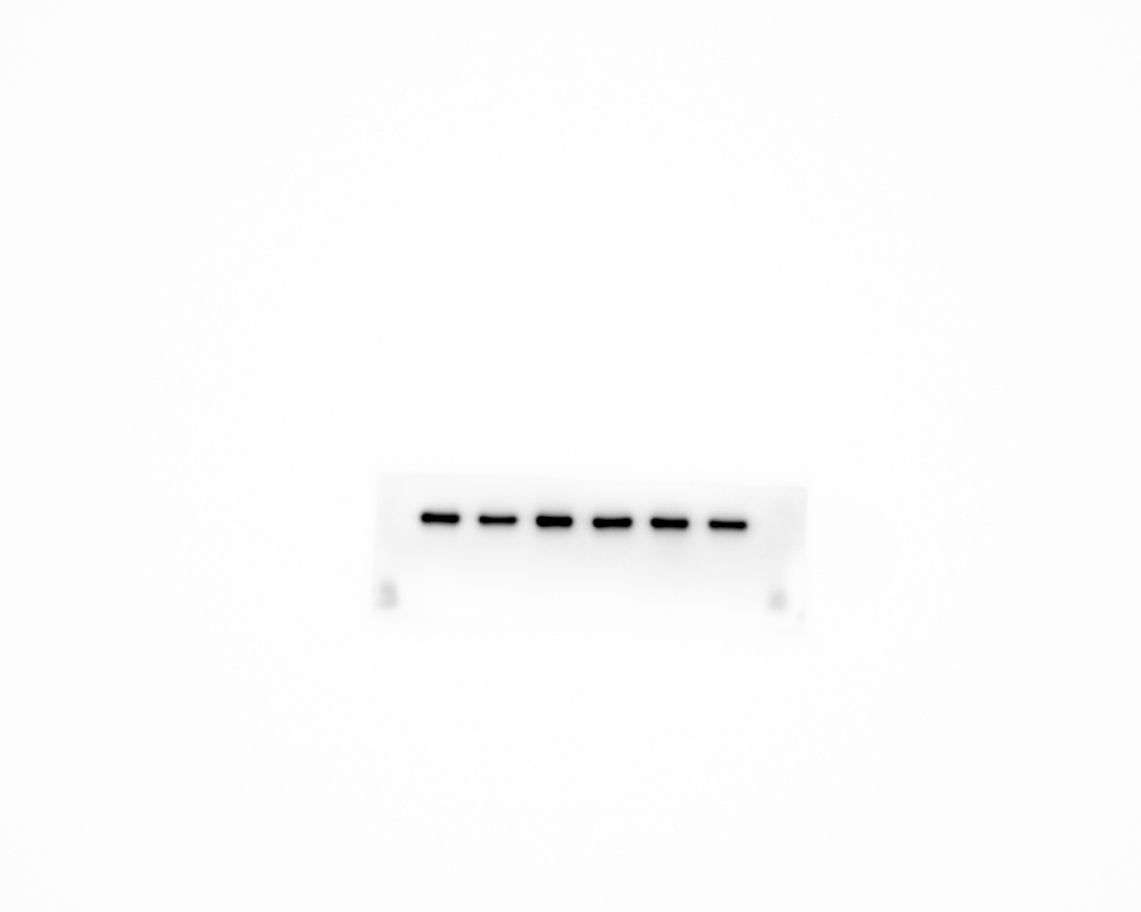


Bcl-2


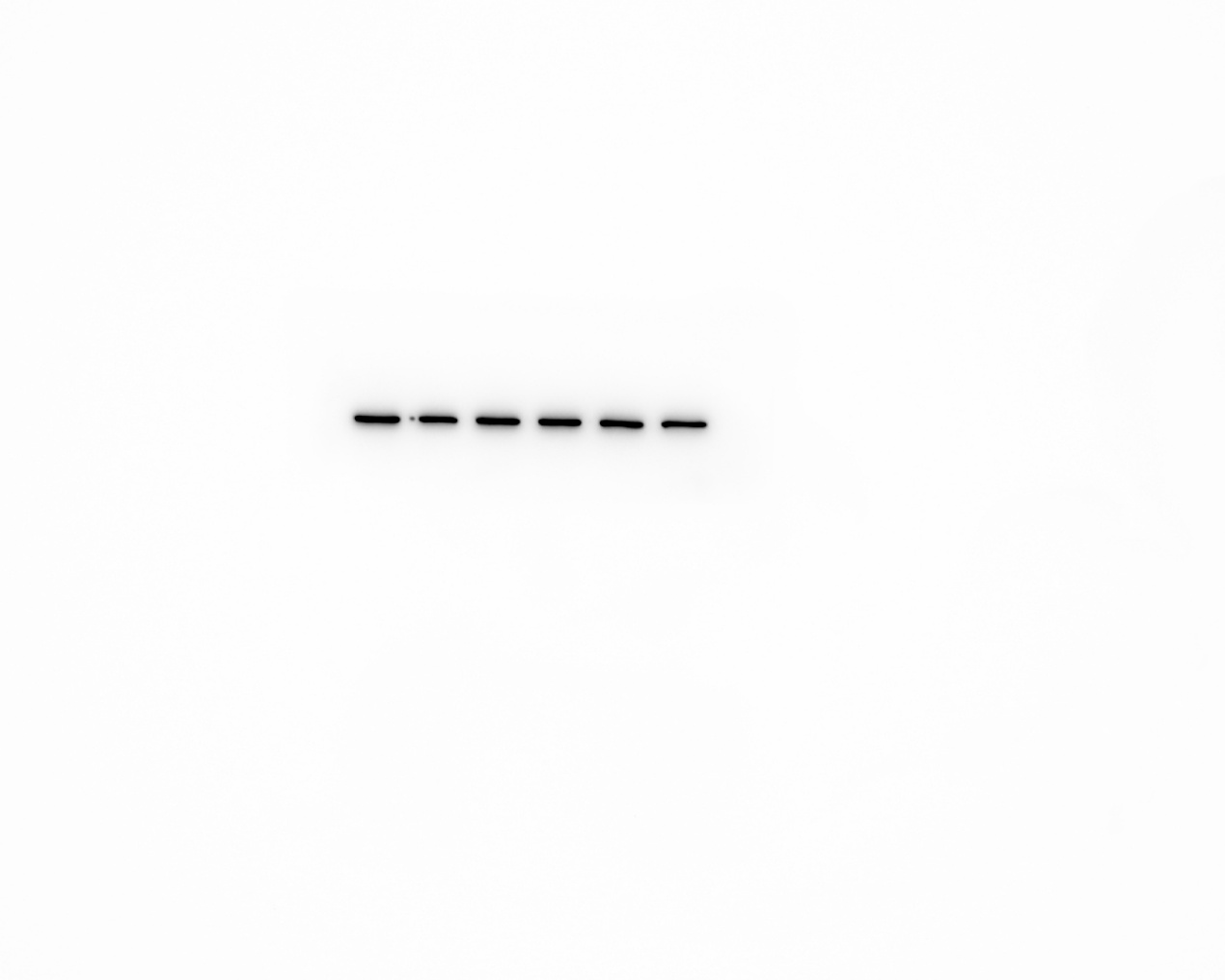


β-actin


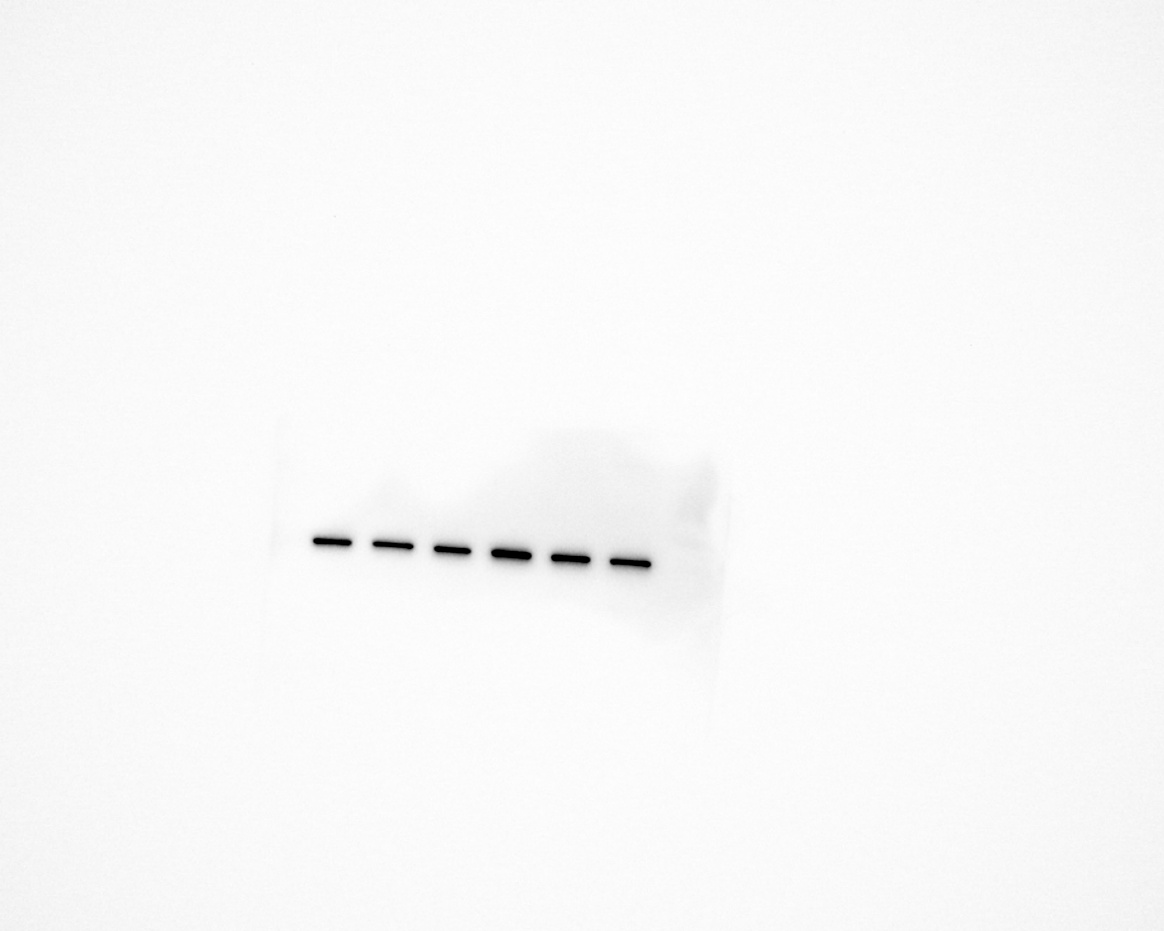

Supplement: Supplementary file 1 [file DataSheet1.docx]
